# Diethyldithiocarbamate-copper nanocomplex reinforces disulfiram chemotherapeutic efficacy through light-triggered nuclear targeting

**DOI:** 10.7150/thno.45558

**Published:** 2020-05-16

**Authors:** Liting Ren, Wenya Feng, Jie Shao, Juan Ma, Ming Xu, Ben-Zhan Zhu, Nanfeng Zheng, Sijin Liu

**Affiliations:** 1 State Key Laboratory of Environmental Chemistry and Ecotoxicology, Research Center for Eco-Environmental Sciences, Chinese Academy of Sciences, Beijing, 100085, China.; 2 State Key Laboratory for Physical Chemistry of Solid Surfaces, Collaborative Innovation Center of Chemistry for Energy Materials, and National & Local Joint Engineering Research Center for Preparation Technology of Nanomaterials, College of Chemistry and Chemical Engineering, Xiamen University, Xiamen, 361005, China.; 3 University of Chinese Academy of Sciences, Beijing, 100049, China.

**Keywords:** diethyldithiocarbamate, DSF-Cu nanocomplex, light-triggered nuclear targeting, metastasis, epithelial-mesenchymal transition

## Abstract

To circumvent the huge cost, long R&D time and the difficulty to identify the targets of new drugs, repurposing the ones that have been clinically approved has been considered as a viable strategy to treat different diseases. In the current study, we outlined the rationale for repurposing disulfiram (DSF, an old alcohol-aversion drug) to treat primary breast cancer and its metastases.

**Methods:** To overcome a few shortcomings of the individual administration of DSF, such as the dependence on copper ions (Cu^2+^) and limited capability in selective targeting, we here artificially synthesized the active form of DSF, diethyldithiocarbamate (DTC)-Cu complex (CuET) for cancer therapeutics. To achieve a greater efficacy *in vivo*, smart nanomedicines were devised through a one-step self-assembly of three functional components including a chemically stable and biocompatible phase-change material (PCM), the robust anticancer drug (CuET) and a near-infrared (NIR) dye (DIR), namely CuET/DIR NPs. A number of *in vitro* assays were performed including the photothermal efficacy, light-triggered drug release behavior, nuclear localization, DNA damage and induction of apoptosis of CuET/DIR NPs and molecular mechanisms underlying CuET-induced repression on cancer metastatic behaviors. Meanwhile, the mice bearing 4T1-LG12-drived orthotopic tumors were employed to evaluate *in vivo* biodistribution and anti-tumor effect of CuET/DIR NPs. The intravenous injection model was employed to reflect the changes of the intrinsic metastatic propensity of 4T1-LG12 cells responding to CuET/DIR NPs.

**Results:** The rationally designed nanomedicines have self-traceability for bioimaging, long blood circulation time for enhanced drug accumulation in the tumor site and photo-responsive release of the anticancer drugs. Moreover, our data unearthed that CuET/DIR nanomedicines behave like “Trojan horse” to transport CuET into the cytoplasm, realizing substantial intracellular accumulation. Upon NIR laser irradiation, massive CuET would be triggered to release from the nanomedicines and reach a high local concentration towards the nucleus, where the pro-apoptotic effects were conducted. Importantly, our CuET/DIR nanomedicines revealed a pronounced capability to leash breast cancer metastases through inhibition on EMT. Additionally, these nanomedicines showed great biocompatibility in animals.

**Conclusion:** These combined data unearthed a remarkably enhanced tumor-killing efficacy of our CuET nanomedicines through nuclear targeting. This work may open a new research area of repurposing DSF as innovative therapeutic agents to treat breast cancer and its metastases.

## Introduction

The cancer occurrence and death are still rising worldwide due to numerous intertwined socioeconomic reasons 1, and comprehensive cancer prevention and control strategies and effective therapeutics are emerging to tackle even greater challenges than before 2-4. Thus far, chemotherapy still leads the most prevalent modalities in cancer treatment 5,6, but this type of therapeutics is greatly undermined by drug resistance, limited targeting to diseased sites and detrimental effects on normal cells 7-9. Under this setting, continuous efforts are being invested in search of new drugs. However, a few factors, including huge cost, long R&D time and slim odds of success, incredibly hinder the successful applications of new drugs. To circumvent these obstacles, drug repurposing, also called drug repositioning or reformulation, has been popularly proposed to treat cancers with the drugs that have been clinically approved to treat other disorders and pathological conditions 10-14. For instance, a number of repurposed anticancer drugs have recently been defined and tested, such as disulfiram (DSF), metformin and statins 15-21.

DSF is an established FDA-approved agent to treat alcoholism dependent on its activity to inhibit acetaldehyde dehydrogenase (ALDH) for decades 22. Preclinical studies manifested a surprisingly encouraging activity against cancers through inhibition on the proteasome activity, pro-apoptotic effects, depletion of cancer stem cells and repression on epithelial-mesenchymal transition (EMT) 23-28. With respect to the molecular mechanisms, in addition to a direct suppression on ALDH enzymatic activity, diethyldithiocarbamate (DTC), a metabolized monomer from DSF, forms DTC-copper (Cu^2+^) complex (CuET), which displays even more potent anticancer efficacy than the parental DSF. Mechanistically, CuET tightly targets nuclear protein localization-4 (NPL4), a crucial adaptor of p97 segregase, leading to prompt protein aggregate formation and resultant heat-shock response associated with oxidative stress and cell death 29. To this end, it would be speculated that Cu ions are indispensable for reinforced DSF chemotherapeutic activity through the formation of CuET complex.

Nevertheless, many questions should be addressed in developing DSF regimes in cancer treatment, as follows. (i) An adequate Cu ion level is a prerequisite for providing substrates to form the CuET complex; however, the intrinsic anfractuous Cu biodistribution and homeostasis of in the human body is not fully understood 30. In other words, DSF-based strategies may be restrained by insufficient Cu ions in tumor microenvironment. (ii) Copper is an essential micronutrient necessary for all types of cells. Based on the CuET formation mechanism, it would be plausible to see the formation of CuET in normal tissues and cells, resulting in unintended side effects and even toxicity. Thus, increasing selective tumor delivery efficacy would be more advisable. (iii) Given that the threshold in finetuning copper metabolism between normal cells and cancer cells is not known till now and excessive Cu^2+^ mass would cause additional oncogenic influences 31,32, extra Cu^2+^ supply in patients without copper deficiency would incur more problems 33-35.

To conquer these above-mentioned obstacles, in the current study, we artificially synthesized CuET/DIR NPs to realize substantial intracellular accumulation, where massive CuET would be triggered to release from the nanomedicines upon near-infrared (NIR) laser irradiation to reach a high local concentration towards the nucleus. Our combined data unraveled a new target of CuET: cellular nucleus, associated with DNA damage and cell death, and pinpointed a promising strategy in developing controllable photosensitive CuET/DIR nanomedicines for greater therapeutics against breast cancer and its metastasis.

## Materials and Methods

### Reagents and chemicals

Copper (II) chloride (CuCl_2_), sodium diethyldithiocarbamate (Na[S_2_CNC_4_H_10_]) and disulfiram (DSF) were obtained from the Sinopharm Chemical Reagent Co. Ltd. (Shanghai, China) and the J&K (Beijing, China), respectively. Lauric acid (99% purity), stearic acid (>97% purity), 1,2-distearoyl-sn-glycero-3-phosphoethanolamine-N-[methoxy(polyethylene glycol)-5000] (Mw=5000, DSPE-PEG5000) and soybean lecithin (Mw≈750) were purchased from the Innochem (Beijing, China), the Acros (Beijing, China), the Laysan Bio (Shanghai, China) and the TCI (Shanghai, China), respectively. DIR iodide (DIR, >98% purity) was purchased from the Sigma-Aldrich (Shanghai, China). Thiazolyl blue tetrazolium bromide (MTT), methanol (CH_3_OH), ethanol (CH_3_CH_2_OH), chloroform (CHCl_3_) and dimethyl sulfoxide (DMSO) were obtained from the Sigma-Aldrich. Hoechst 33342, LysoTracker Green DND-26, culture medium, fetal bovine serum (FBS) and phosphate buffered saline (PBS, 10 mM, pH=7.4) were purchased from the Thermo Fisher Scientific (Beijing, China).

### Synthesis of CuET

In brief, CuCl_2_ (1 mmol, 135 mg) and sodium diethyldithiocarbamate (Na[S_2_CNC_4_H_10_]) (2 mmol, 342 mg) were separately dissolved in deionized water. The metal ion solution was mixed with the dissolved diethyldithiocarbamate ligand solution with a ratio of 1:2 at the molar concentration. The products were immediately formed as precipitates, followed by centrifugation and re-dissolution in chloroform. The molecular precursors were recrystallized by diffusing ethanol into the above chloroform solution in 1 week prior to the following experiments.

### Synthesis of CuET/DIR NPs and CuET/Cy5.5 NPs

Phase-change materials (PCM) NPs were fabricated following a previously reported method with minor revisions 36. Briefly, lauric acid and stearic acid (4:1 by weight) were dissolved in methanol at 4 mg/mL. In order to enhance the stability of NPs and the dispersion in biological fluids, the hydrophilic polymeric shell with antibiofouling properties was modified onto the surface of PCM NPs. Lecithin and DSPE-PEG5000 (3:1 by weight) were dissolved in 4% aqueous ethanol at 1 mg/mL. Phospholipid solution (3 mL) was heated to 50 °C for 30 min. The PCM solution (600 μL) mixed with the desired payloads (48 μL 2.5 mg/mL DIR or Cy5.5 in DMSO and/or 48 μL 2.5 mg/mL CuET in DMSO) was then added dropwise into the preheated phospholipid solution, followed by vigorous vortex for 3 min. After cooling down in the ice water for 2 min, the cloudy solution was warmed up to ambient temperature in 2 h, and was then vortexed for 2 min, followed by filtration through a 0.2 µm surfactant-free cellulose acetate membrane (Thermo Fisher Scientific). The unencapsulated molecules and organic solvents were removed using a VIVASPIN 6 centrifugal concentrator (Sartorius, MWCO=10 kDa). After washing with water for 3 times, the resultant nanoparticles were suspended in water for further use.

### Characterization of CuET/DIR NPs

PCM NPs were negatively stained with 1.5% phosphotungstic acid for transmission electron microscope (TEM) characterization (H-7500, Japan). Scanning electron microscope (SEM) imaging of the NPs was performed on a field-emission SEM (NOVA NANOSEM430, FEI, Netherlands) at 5-10 kV after gold coating for 120 s (EM-SCD500, Leica, Germany). The particle size distribution was measured at 25 °C by dynamic light scattering (DLS, Zetasizer Nano ZS, Malvern). The UV-vis absorption spectra were recorded on a UV-vis spectrophotometer DU-800 (Backman, USA). The concentration of DIR was assayed on a microplate reader (Thermo Fisher Scientific, Beijing) for fluorescence measurement at 780 nm with CuET/DIR NPs dissolved in methanol. The concentration of CuET was determined by inductively coupled plasma mass spectrometry (ICP-MS) (Agilent 8800, Japan) based on Cu after nitrification with 1.5% HCl and 0.5% HNO_3_. Drug encapsulation efficiency (EE) was calculated using the following equation:

EE=(weight of the drug in nanoparticles)/(weight of the added drug)×100%

### Photothermal efficacy of CuET/DIR NPs

The aqueous solution of CuET/DIR NPs (1 mL, [DIR]=2.0 μg/mL) was subjected to 808 nm laser irradiation at different power density for 10 min, and the temperature was recorded at intervals of 30 s with thermocouple (TES 1315, TES Electrical Electronic Corp., China). After the temperature dropped to the ambient temperature, 2 additional cycles of irradiation were performed with each cycle lasting for 10 min.

### NIR-triggered drug release

To measure the release profile of CuET under NIR laser irradiation, the aqueous solution of CuET/DIR NPs (1 mL, [CuET]=0.4 μg/mL) was irradiated under 808 nm laser at a power density of 2 W/cm^2^ for 5 min. At indicated time points, the irradiated solution (20 μL) was retrieved and mixed with water (80 μL) for fluorescence measurement using a microplate reader at 432 nm (Thermo Fisher Scientific, Beijing). For the heating/cooling experiment, the aqueous suspension of CuET/DIR NPs (1 mL) was repeatedly irradiated under 808 nm laser at a power density of 2 W/cm^2^ for 5 min, along with an interval of 5 min for heat dissipation. The percentage of CuET release was calculated by (I_t_-I_0_)/(I_100_-I_0_)×100, where I_t_ is the fluorescence intensity of the solution at the indicated time points, I_0_ is the initial fluorescence intensity of the solution prior to laser irradiation, and I_100_ is the fluorescence intensity of CuET at the initial concentration.

### Cell culture and animal models

Here, breast cancer cells MCF-7, 4T1 and 4T1 subline 4T1-LG12 cells were cultured in RPMI 1640 medium supplemented with 10% FBS and 1% antibiotics (containing penicillin and streptomycin) in a humidified incubator at 37 °C containing 5% CO_2_. All animal experiments were approved by the Animal Ethics Committee at the Research Center for Eco-Environmental Sciences, Chinese Academy of Sciences. Female mice (7-8 weeks old with BALB/c genetic background) were purchased from the Vital River Laboratory Animal Technology Co. Ltd (Beijing, China). The orthotopic model (under the skin at the mammary fat pad sites) and the intravenous injection model were accordingly established following the instructions in our previous reports 37,38. The NIR laser irradiation was only implemented at solid tumors *per se*. Because 4T1-LG12 cells were genetically integrated an exotic luciferase gene, metastatic tumors were visualized and monitored through the bioluminescent signals after injection of 100 μL luciferin (15 mg/mL) using an IVIS Spectrum Imaging System (Caliper Life Sciences Inc., USA). In the orthotopic model, when the volume of primary tumors reached approximately 150 mm^3^, mice with a comparable volume of primary tumors were randomly divided into different groups for following treatments. Tumor volume was measured by a caliper and calculated according to the following equation: tumor volume=(length×width^2^)/2. To assess lung metastasis, 4T1-LG12 cells was first pre-treated with CuET or CuET/DIR NPs together with NIR laser irradiation for 24 h, and pre-treated cells were afterwards intravenously injected into mice through tail vein. Lung metastasis was eventually examined when mice were sacrificed.

### Tissue distribution of CuET/DIR NPs in mice

Mice bearing 4T1-LG12-drived orthotopic tumors were intravenously injected with CuET/DIR NPs (432 μg/Kg body weight based on CuET) *via* tail vein. After imaging of the whole animals, primary tumors together with other organs were dissected for imaging at different time points (n=5 for each point). Collected tumors and organs were weighed, and then dissolved in acid solution containing 1.5% HCl and 0.5% HNO_3_ for nitrification at 65 ℃. The Cu content in tumors and organs were analyzed by ICP-MS.

### Histological examination

Tissue specimens were collected and fixed in 4% PBS-buffered paraformaldehyde, and tissue sections were stained with hematoxylin and eosin (H&E) staining following the standard protocols, followed by examination under an optical microscope (Axio Scope A1, Carl Zeiss, Inc., Germany).

### Fluorescent microscopy characterization

4T1-LG12 cells were seeded in confocal dishes at a density of 3-4×10^5^ cells per dish and were then cultured overnight. CuET/Cy5.5 NPs were added into the cells and incubated at 37 °C for 3 h. After washing with PBS, cells were stained with Hoechst 33342 (1 mL, 10 μg/mL) and Lysotracker Green DND-26 (1 mL, 250 nM) in RPMI-1640 at 37 °C for 20 min. After washing with PBS for 3 times, the fresh culture medium was supplemented for fluorescence microscopy imaging (DMI6000, Leica).

### *In vitro* cytotoxicity and cellular uptake assessments

Cells were seeded in 96-well plates at a density of 1-1.5×10^4^ cells per well and were then cultured overnight, followed by different treatments. With respect to cells receiving NIR laser irradiation, cells were irradiated with the 808 nm laser at a power density of 2 W/cm^2^ for 5 min after incubation with different treatments for 12 h, followed by additional 12 h culture. Post treatments, cell viability was determined through by Cell Counting Kit-8 (CCK-8) assay following a standard protocol (Solarbio, 1000 T, China). After various treatments, cell death was determined using PI staining through flow cytometry analysis (FACS Calibur, BD Biosciences, CA). To reflect the cellular uptake of CuET and CuET/DIR NPs, treated cells were collected for ICP-MS determination of Cu mass.

### Transwell invasion/migration and wound-healing assays

Regarding the transwell invasion/migration assay, matrigel (200 μg/mL in 50 μL) was first loaded onto the upper chambers of transwells, and then incubated at 37 °C for 2 h. Next, 5.0×10^4^ cells with or without treatments were seeded onto the upper chambers. Thereafter, culture medium with 10% FBS (in 100 μL), acting as the chemoattractant, was added into bottom wells for consecutive culture for 24 h. Then, the filters were removed from the upper chambers, and fixed with 4% formaldehyde. The filters were stained with 1% crystal violet, followed by examination under an optical microscope. In the meanwhile, the wound-healing assay was carried out following the method, as described previously 39.

### Western blot analysis

Harvested cells were lysed in RIPA buffer containing protease inhibitor cocktail (Roche Diagnostics, USA). Concentrations of total cellular proteins were assayed using the BCA method (Solarbio, China). An equal amount of total proteins for each group were subjected to SDS-PAGE and thereafter transferred onto nitrocellulose membranes. Immunoblotting was carried out following a standard protocol, as previously described 40. Primary antibodies (Abs) were as follows, anti-β-actin Ab (1:1000 dilution, Proteintech, USA), anti-GAPDH Ab (1:1000 dilution, Proteintech, USA), anti-Vimentin Ab (1:1000 dilution, Proteintech, USA), anti-β-catenin Ab (1:1000 dilution, Proteintech, USA), anti-occludin Ab (1:1000 dilution, Proteintech, USA), anti-γ-H2AX Ab (1:1000 dilution, Proteintech, USA) and anti-cleaved caspase-9 Ab (1:1000 dilution, Proteintech, USA).

### Cell nucleus extraction

Nuclei from 4T1-LG12 cells after treatments were collected with a Nuc-Cyto-Mem preparation kit (Applygen Technologies Inc, China). Briefly, cytosol extraction reagent (500 μL) was added to the precipitate per 1×10^7^ cells for resuspension, followed by ice bath for 2 min. The cell suspension was transferred to a precooled glass homogenizer and homogenized manually in ice bath for 20-30 times. The crude cell nuclei were collected by centrifugation at 800 g and 4 °C for 5 min. Then, nucleus extraction reagent (500 μL) was added to the crude cell nuclei for resuspension, followed by centrifugation at 4,000 g and 4 °C for 5 min. After resuspension with nucleus extraction reagent and centrifugation for 2 times, the precipitate was resuspended in suspension buffer (50 μL) for the next-step analysis of ICP-MS.

### DNA damage determination through γ-H2AX immunofluorescence analysis

4T1-LG12 cells were seeded in confocal dishes at a density of 5×10^4^ cells for 24 h. The experiment was divided into 4 groups (Control, CuET at 0.1 μM, CuET at 1 μM, CuET/DIR NPs + NIR containing 1μM CuET). Next, the last group was subjected to irradiation with NIR laser (808 nm, 2 W/cm^2^ lasting for 5 min) after incubation with CuET/DIR NPs (containing 1μM CuET, 1 mL) for 3 h, followed by additional 3 h culture. Afterwards, cells were fixed with 4% paraformaldehyde for 15 min, rinsed 3 times with PBS, and then permeabilized with triton-X 100 for 10 min at room temperature, followed by washing with PBS. Thereafter, cells were exposed to blocking buffer (1% BSA in PBS) for 1 h at room temperature, and then incubated with γ-H2AX Ab (dilution 1:1000) overnight at 4 ℃. On the next day, after washing, the cells were incubated with Cy3-conjugated goat anti-rabbit IgG (H+L) (dilution 1:1000) for 1 h at the room temperature. Moreover, nuclei were stained with DAPI at room temperature. At last, the cells were imaged under a confocal microscope (DMI6000, Leica).

### DNA extraction and enzymatic digestion

Genomic DNA was extracted from the harvested cells using a Genomic DNA Purification Kit (Solarbio, China) according to the manufacturer's instructions. The concentration and quality of the extracted DNA were evaluated by measuring the absorbance at 260 nm and 280 nm. The extracted DNA was digested with 1 U DNase I, 2 U calf intestinal phosphatase and 0.005 U snake venom phosphodiesterase I (New England Biolabs, Ipswich, MA, USA) at 37 °C for 24 h. Proteins from the DNA digestion system were removed prior to the ultra-performance liquid chromatography-tandem mass spectrometry (UPLC-MS/MS) analysis, using the microcon centrifugal filter device (Millipore, Bedford, MA, USA) with the 3,000 D cutoff membrane through centrifugation at 10,000 g for 30 min.

### UPLC-SRM MS/MS analysis

The UPLC separation was conducted on a Thermo TSQ Quantum Access Max equipped with Accela U-HPLC and autosampler (Thermo Fisher Scientific, Waltham, MA). A reversed-phase Hypersil GOLD column (100×2.1 mm, 1.9 μm, Thermo) was used, and 95% water (containing 0.1% formic acid) and 5.0% methanol was used as mobile phase at a flow rate of 0.2 mL/min. The eluate from the HPLC column was directly introduced into an ESI-triple quadrupole mass spectrometer (TSQ Quantum Access MAX). The mass spectrometer was operated in the positive ion mode. For Selective Reaction Monitoring (SRM) analysis, collision energy was performed at 15 eV. The fragmentor voltage was 90 V, and nitrogen was used as nebulizer gas. The desolvation gas (nitrogen) was heated to 300 °C and delivered at a flow rate of 9.0 L/min. The capillary voltage was set at 3,500 V. The injection volume is 5.0-15.0 μL for the digested DNA. Then, 8-oxo-7,8-dihydro-2'-deoxyguanosine (8-oxodG) in cellular DNA was detected in the form of the mononucleotide 8-oxodG by monitoring the transitions of m/z 284.1→168.1. The amount of 8-oxodG was calibrated by the standard curve.

### Statistical analysis

The statistical analysis of experimental data was carried out using independent *t-test* or one-way ANOVA test with the SPSS Statistics 17.0 software. All experimental data are presented as mean ± standard error. Statistical significance was determined with *: P < 0.05 and #: P < 0.001.

## Results and Discussion

**Figure 1:**
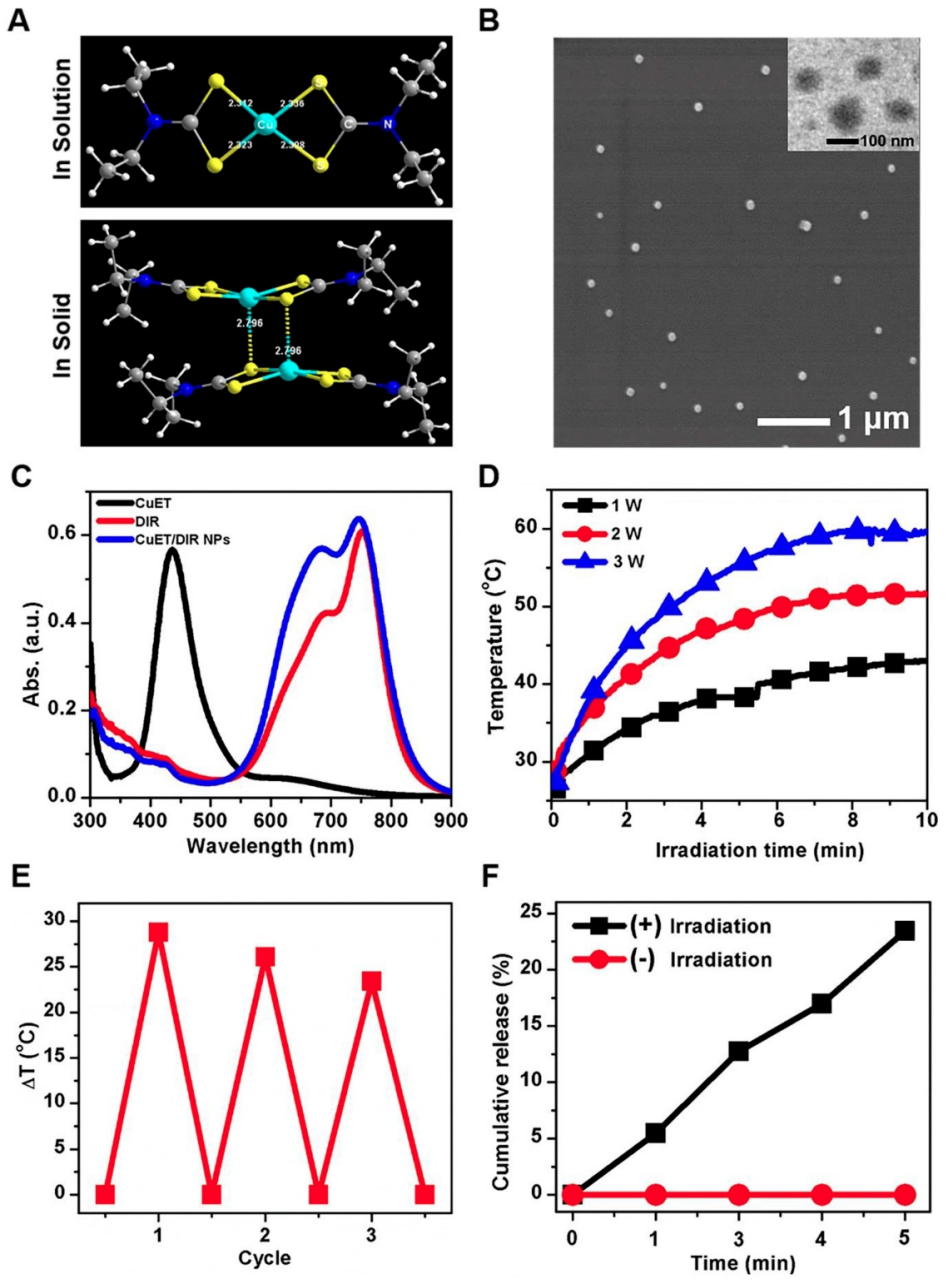
** Characterization and photothermal efficacy of CuET/DIR NPs.** (A) Molecular structures of CuET monomer and dimer in solution and solid, suggesting rich electronic density on the sulfur atom within CuET. (B) Representative SEM and TEM images of CuET/DIR NPs. The TEM image of CuET/DIR NPs is shown in the upper-right insert. (C) UV-vis absorption spectra of free CuET (black), free DIR (red) and CuET/DIR NPs (blue). (D) Temperature elevation of CuET/DIR NPs solution (with 2.0 μg/mL DIR) under 808 nm laser irradiation at different power density for 10 min. (E) Change of the photothermal heating behaviors for CuET/DIR NPs after repeated cycles of 808 nm laser irradiation at a power density of 2 W/cm^2^. (F) Cumulative CuET release from CuET/DIR NPs under laser irradiation over time (808 nm, 2 W/cm^2^).

To obtain the active form of DSF, we deliberately synthesized CuET complex, as described previously 41. As shown in Figure 1A, the molecular structure of CuET was verified by X-ray single-crystal diffraction, with characteristic absorption peak at 432 nm (Supplementary Figure S1). Herein, synthesized CuET complex was used in the following mechanistic investigations. For even though direct administration of CuET is an option to enhance the anticancer efficacy of DSF, unsatisfactory drug delivery to selective sites remains a large challenge for CuET, which hinders its clinical applications 42-44. To address this challenge, we loaded CuET into nano-vehicles, organic phase-change nanomaterials (PCMs), to obtain PCM@CuET NPs. To fabricate PCMs, a eutectic mixture of lauric acid and stearic acid was used as the core, and phospholipids served as the shell. As shown in the TEM image (Figure 1B, the insert), the PCM@CuET NPs displayed uniform sphere-like morphology with the diameter in the range of 80-120 nm. The average hydrodynamic diameter of PCM@CuET NPs was about 123 nm, as determined by DLS, and the zeta potential of PCM@CuET NPs was -20.8 mV (Supplementary Figure S2).

To build up a smart release system, in addition to CuET, a photochemical agent DIR was also encapsulated into PCM NPs, in that DIR would sensitively respond to NIR laser. As reflected by the characteristic absorption spectra (Figure 1C), successful loading of CuET and DIR was substantiated 36, with an encapsulation efficiency as high as 12% and 62%, respectively. It is worth noting that the absorption peak of DIR was slightly broadened after encapsulation into PCM (Figure 1C), likely due to the hydrophobic interaction between DIR and PCM. In addition, the NPs in different media, such as water, PBS and DMEM, manifested good dispersion stability for at least 1 week without visible precipitation. Thereafter, the photothermal properties of PCM@CuET@DIR NPs (denoted as CuET/DIR NPs here) were assessed. Figure 1D presents the photothermal heating curves showing a strong power-dependent temperature increase with the maximum temperature up to 51.6 ℃ at a power density of 2 W/cm^2^ for CuET/DIR NPs at 2.0 μg/mL based on DIR. Besides, the photothermal heating curves were tested at different concentrations based on DIR with a power density of 2 W/cm^2^. As shown in Supplementary Figure S3, a concentration-dependent temperature increased for CuET/DIR NPs. The photothermal stability assessment unveiled that the CuET/DIR NPs still bore pronounced photothermal efficacy after 3 repeated cycles of laser irradiation (Figure 1E). Subjected to NIR laser irradiation, the drug release from CuET/DIR NPs was evaluated. As shown in Figure 1F, CuET was gradually dissolved from CuET/DIR NPs over time in response to NIR laser irradiation, in contrast to no drug release in the control samples without NIR laser irradiation, indicating temperature-induced phase change of PCM and resultant release of CuET upon the photothermal heating of DIR. Moreover, photothermal-controlled drug release was determined during heating/cooling cycles. Supplementary Figure S4 shows a steady and long-term feature for the release of CuET. In agreement with our data, a previous study manifested a similar release profile for 30 min 36. Further DLS and SEM measurements indicated that the average diameter slightly increased to 134 nm (Supplementary Figure S5A-B), presumably being attributable to the melting of PCM and consequent particle agglomeration. Together, we here made stable temperature-responsive CuET nano-carriers for anti-cancer activity tests.

**Figure 2:**
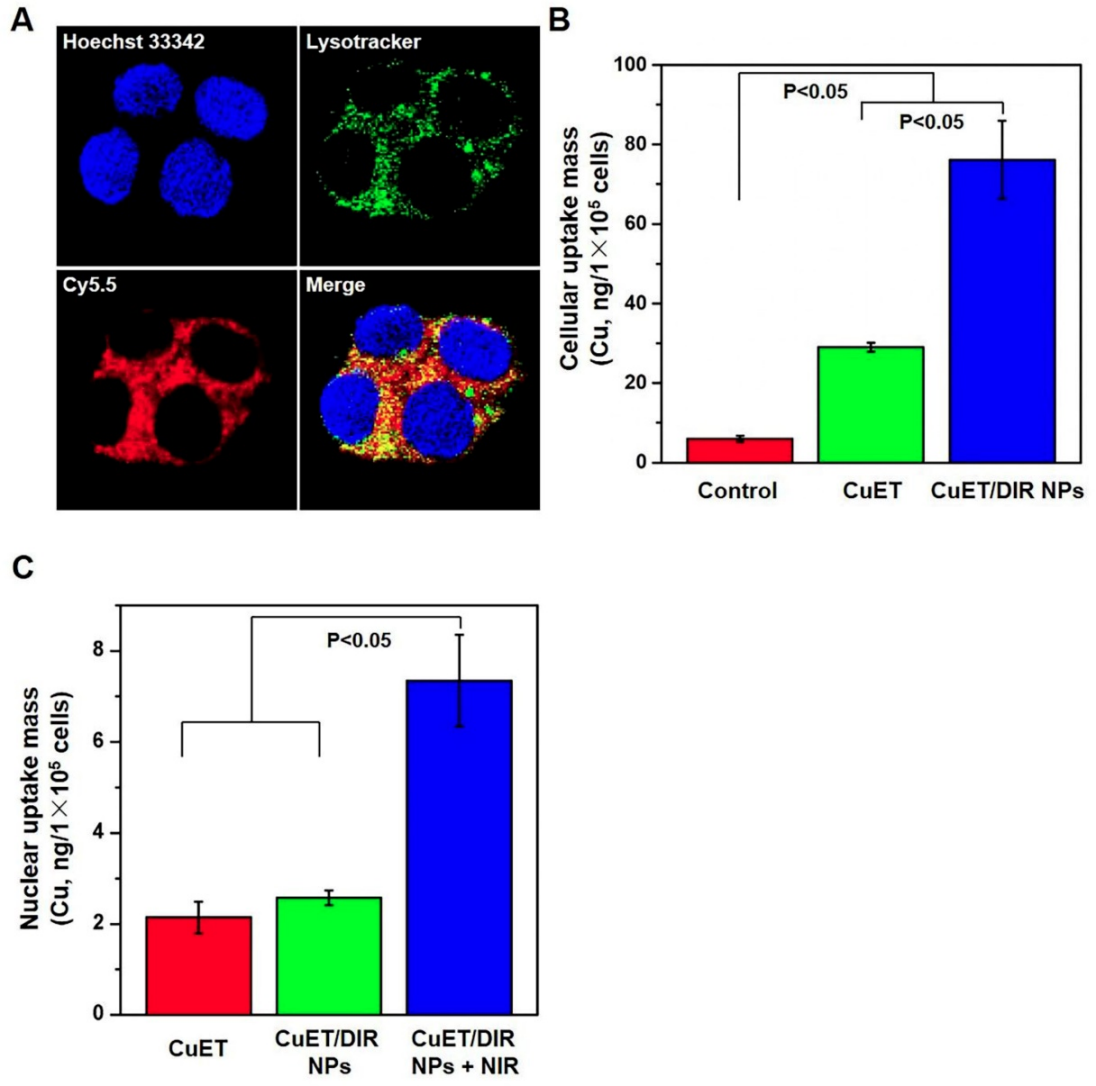
** Nuclear localization of CuET/DIR nanomedicines.** (A) Confocal microscopy images showing the locations of CuET/Cy5.5 NPs (with 0.4 μg/mL CuET and 2.0 μg/mL Cy5.5) in 4T1-LG12 cells after incubation for 3 h, with Hoechst 33342 (blue), Lysotracker (green) and Cy5.5 (red). (B) Quantitative analysis of intracellular Cu content in 4T1-LG12 cells upon CuET and CuET/DIR NPs at the same dose of CuET (1 μM) for 24 h by ICP-MS (n=3). (C) Quantitative analysis of nuclear Cu content in 4T1-LG12 cells upon CuET and CuET/DIR NPs together with/without NIR laser irradiation (808 nm, 2 W/cm^2^ for 5 min) at the same dose of CuET (1 μM) for 24 h through ICP-MS (n=3).

Thereafter, we attempted to look for new targets of CuET, except p97 segregase adaptor: NPL4, as recently discovered 29. Recent studies reported elevated radiosensitivity of glioblastoma cells with DSF pretreatment through DNA damage, cell cycle interruption and finally apoptosis 45,46, implying likely localization of CuET towards nuclei. To this end, its subcellular localization was closely probed. Since no fluorescence could be used to visualize CuET/DIR NPs inside cells, we deliberately labelled CuET/DIR NPs with the Cy5.5 dye. As shown in Figure 2A, remarkable co-localization was visualized between Cy5.5 fluorescence (in red) and lysotracker (in green, suggestive of lysosome), revealing that CuET/DIR NPs were engulfed into cells *via* endolysosomal pathway. To confirm cellular uptake of CuET, ICP-MS was performed to measure Cu mass in cells treated with CuET at 1 μM and CuET/DIR NPs (with an equal amount of CuET at 1 μM). Our results showed marked cellular uptake of CuET into 4T1-LG12 cells upon treatment with CuET only, and CuET/DIR NPs further elevated the cellular uptake by approximately 2.3 times compared to cells with only CuET treatment (Figure 2B, P<0.05), suggesting that the NPs in fact favored cellular uptake of CuET. More strikingly, nuclear enrichment of Cu was evidenced by more than 3 times in cells treated with CuET/DIR NPs (with 1 μM CuET) together with 808 nm laser irradiation than those upon CuET alone at 1 μM, unveiling that CuET/DIR NPs together with NIR laser irradiation could further facilitate the transportation of CuET into nuclei (Figure 2C, P<0.05). The finding on nuclear accumulation of CuET has not been recognized before, and pinpoints a new mechanism of CuET-induced cytotoxicity, different from interaction with NPL4. Hereby, we unearthed a nuclear targeting mechanism for CuET nanomedicines, as shown in Supplementary Figure S6. First, CuET nanomedicines behave like “Trojan horse” to realize substantial intracellular accumulation. In support of this statement, as shown in Figure 2A, massive nanomaterials were found to accumulate in the perinuclear region. Second, upon NIR laser irradiation, massive CuET would be triggered to release from the nanomedicines and reach a high local concentration towards the nucleus. In fact, this serves as a prerequisite for further delivering them into the nucleus 47. Third, mild NIR laser could induce local hyperthermia in the perinuclear region 48. Under this context, it could be assumed that, subjected to the effect of local hyperthermia around the perinuclear region, tremendous anticancer drugs would rapidly occupy the nucleus, as proposed previously 47-49.

**Figure 3:**
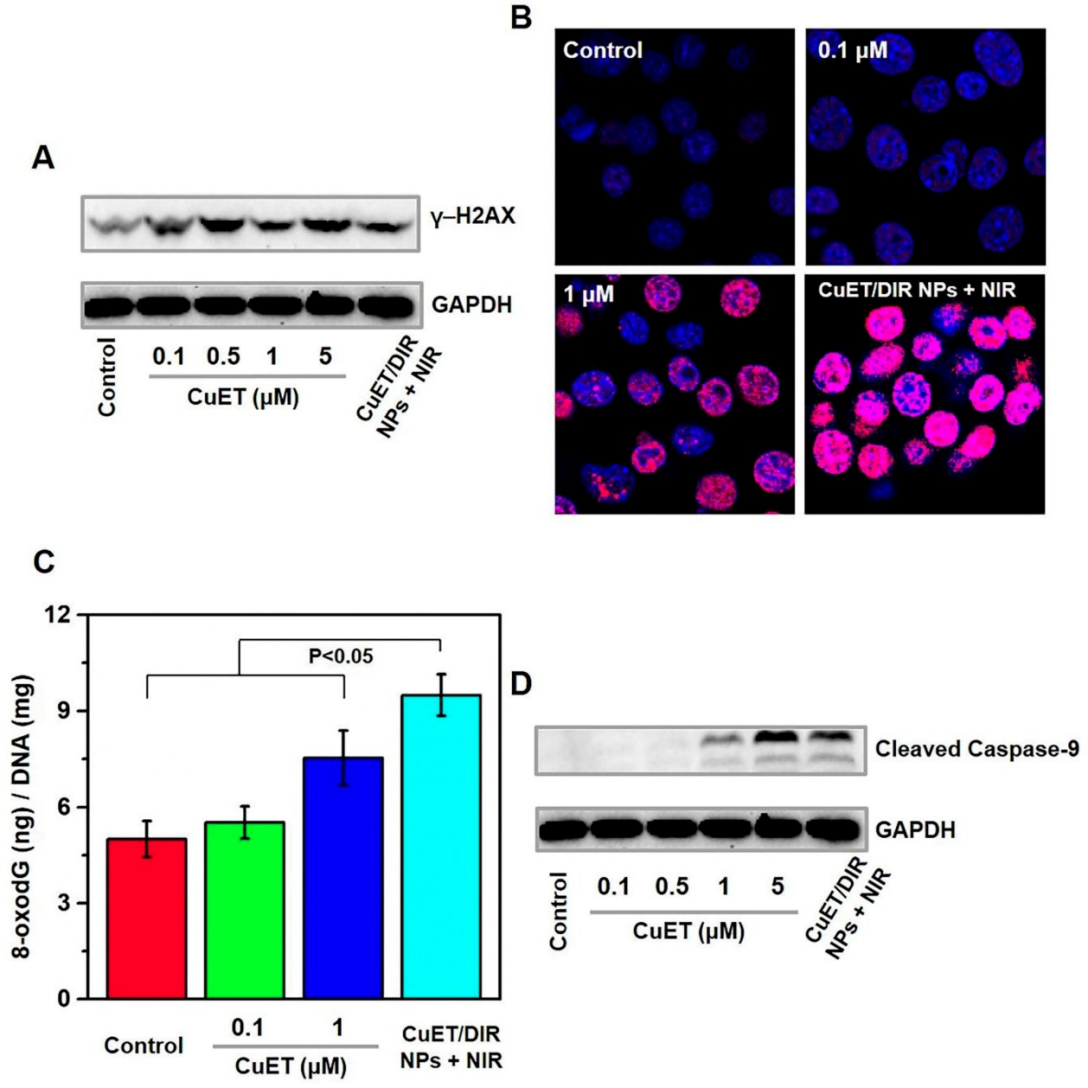
** CuET-induced DNA damage and induction of apoptosis.** (A) Protein levels of γ-H2AX in 4T1-LG12 cells upon CuET at different concentrations and CuET/DIR NPs (containing 1 μM CuET) together with NIR laser irradiation (808 nm, 2 W/cm^2^ for 5 min) for 6 h. (B) Representative immunofluorescent images of γ-H2AX induced by CuET at 0.1 and 1 μM and CuET/DIR NPs (containing 1 μM CuET) in combination of NIR laser irradiation (808 nm, 2 W/cm^2^ for 5 min) for 6 h. DAPI (blue), Cy3 (red) and merged image (pink). (C) The levels of 8-oxodG in 4T1-LG12 cells upon CuET at different concentrations and CuET/DIR NPs (containing 1 μM CuET) with NIR laser irradiation (808 nm, 2 W/cm^2^ for 5 min) for 24 h. (D) Protein levels of cleaved caspase-9 in 4T1-LG12 cells upon CuET at different concentrations and CuET/DIR NPs (containing 1 μM CuET) together with NIR laser irradiation (808 nm, 2 W/cm^2^ for 5 min) for 6 h.

Inspired by the above nuclear localization results, we thereby examined the likely DNA damage and cell death in cells responding to CuET and CuET/DIR NPs. First, a popularly used surrogate to recognize DNA double-strand breaks, γ-H2AX, was assessed. As manifested by Western blotting results (Figure 3A), CuET largely increased the concentration of γ-H2AX in 4T1-LG12 cells at 0.1 to 5 μM. Moreover, CuET with the aid of PCM NPs, namely CuET/DIR NPs, further increased the mass of γ-H2AX under NIR laser irradiation, relative to CuET alone at the same concentration (Figure 3A). This difference was also demonstrated by *in situ* γ-H2AX determination, as much stronger positive signaling (in red) was visualized in CuET/DIR NP-treated cells, compared to CuET-treated cells at 1 μM (Figure 3B), supporting the findings on elevated nuclear delivery CuET by nano-carriers together with NIR laser irradiation (Figure 2C, P<0.05). To corroborate CuET-induced DNA damage, the levels of cellular 8-oxodG, as a sensitive parameter of DNA damage, were measured through UPLC-MS/MS analysis. As shown in Figure 3C, CuET increased the levels of 8-oxodG in 4T1-LG12 cells, especially at 1 μM with more than 50% elevation relative to untreated cells (P<0.05). Moreover, DIR-involved combinatory thermo-chemotherapy induced much greater 8-oxodG/DNA production than CuET alone at the same concentration (Figure 3C, P<0.05), in parallel to the changes of γ-H2AX, as described above. As a result, elevated cell death was found in cells upon CuET treatment at different concentrations, with a substantial PI-positive proportion at 5 μM relative to untreated cells (Supplementary Figure S7). In support of the above findings, the central downstream executor to trigger apoptosis in response to DNA damage, caspase-9 in the activated form (*i.e.* cleaved caspase-9) was increased in CuET-treated cells, and further increased in cells treated with CuET/DIR NPs (containing 1 μM CuET) at the same concentration of CuET together with NIR laser irradiation (Figure 3D). These data together highlighted the reinforced cytotoxicity of CuET in the form of CuET/DIR NPs towards 4T1-LG12 cells due to its nuclear transportation and resultant DNA damage.

**Figure 4:**
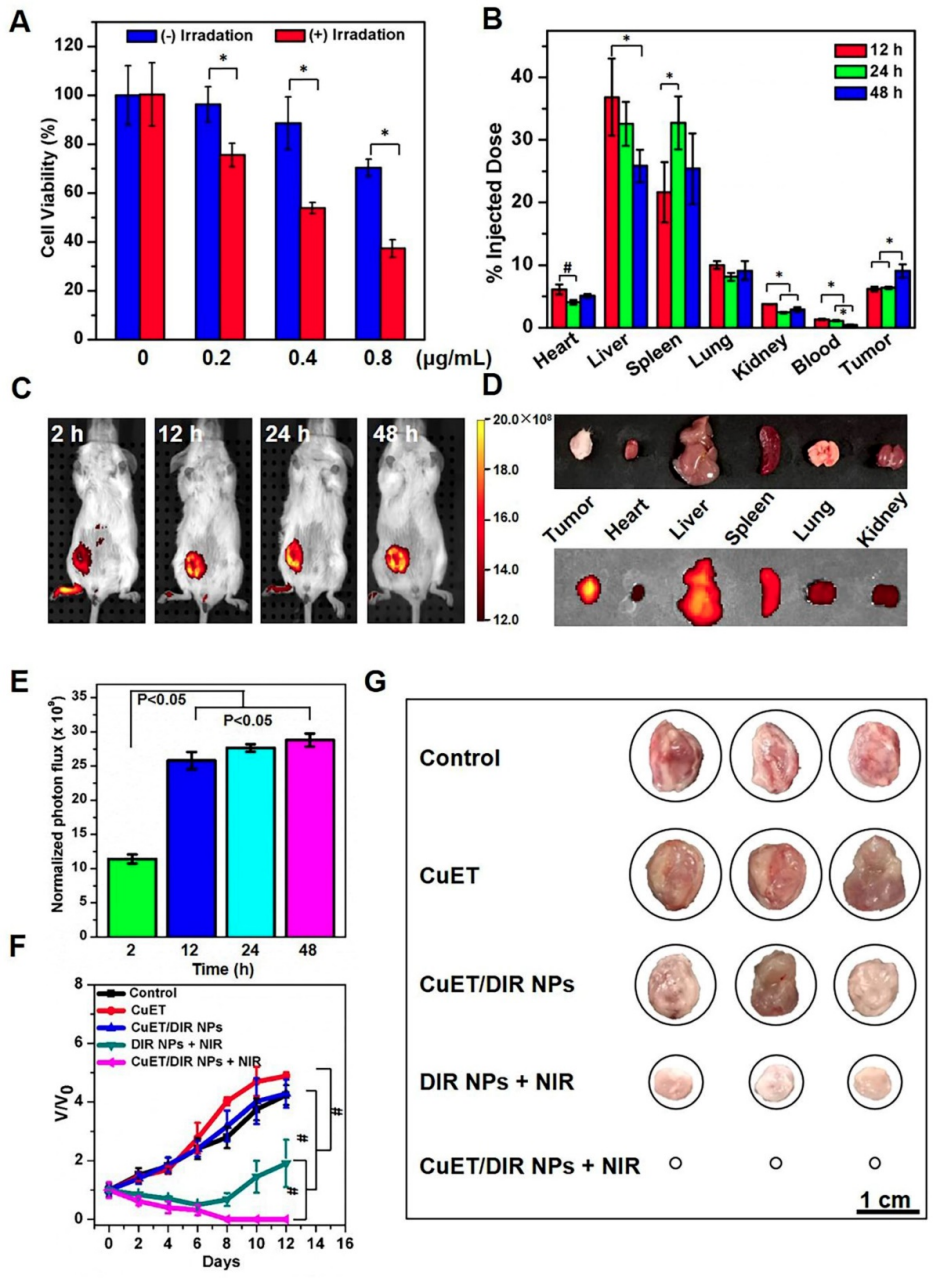
** Tumor localization and tumor-killing assessment.** (A) Phototoxicity towards 4T1-LG12 cells in response to NIR laser irradiation of CuET/DIR NPs at different concentrations, 0.2 μg/mL CuET with 1.0 μg/mL DIR; 0.4 μg/mL CuET with 2.0 μg/mL DIR; 0.8 μg/mL CuET with 4.0 μg/mL DIR, respectively, at a power density of 2 W/cm^2^ for 5 min, followed by additional 12 h culture. *: P<0.05, relative to cells without NIR laser irradiation. (B) Tissue distribution of CuET/DIR NPs in 4T1-LG12 tumor-bearing mice after administration of CuET/DIR NPs at 432 μg/Kg body weight based on CuET through tail vein at different time points. *: P<0.05. (C) *In vivo* fluorescent imaging of mice bearing 4T1-LG12 tumors after tail vein injection of CuET/DIR NPs at 1.2 mg/Kg body weight based on DIR at different time points. (D) *Ex vivo* fluorescent imaging of major organs and tumors dissected from mice after tail vein injection of CuET/DIR NPs at 48 h. (E) Quantification of the bioluminescent signals at the tumor sites at different time points. (F) The growth curves of tumor volumes over time and (G) representative tumor images in different groups with orthotopic implantation of 4T1-LG12 cells at the mammary fat pads (5×10^4^ cells/per mouse, n=5). Different treatments were performed 12 days after the volume of tumors reached 150 mm^3^. CuET group: CuET administration (216 μg/Kg body weight); CuET/DIR NPs group: CuET/DIR NPs administration (216 μg/Kg body weight based on CuET and 1.2 mg/Kg body weight based on DIR); DIR NPs + NIR group: DIR NPs administration (1.2 mg/Kg body weight based on DIR) together with NIR laser irradiation (808 nm, 2 W/cm^2^ for 5 min) 12 h post administration; CuET/DIR NPs + NIR group: CuET/DIR NPs administration (216 μg/Kg body weight based on CuET and 1.2 mg/Kg body weight based on DIR) in combination with NIR laser irradiation (808 nm, 2 W/cm^2^ for 5 min) 12 h post administration. The administration was implemented through tail vein every 48 h for a total of 2 times.

Next, the tumor-killing efficacy was closely assessed for CuET and CuET/DIR NPs in various cancer cells. In analogy to the above findings, as shown in Supplementary Figure S8A, CuET showed remarkable cytotoxicity after treatment for 24 h even at low concentrations from 0.1 to 5 μM in various cancer cell lines (P<0.05), including human breast cancer cell line MCF-7, murine breast cancer cell line 4T1 (parental line) and its aggressive subline 4T1-LG12, which harbors enhanced propensity to metastasize to the lung 37,38. In contrast to little cytotoxicity for DSF and the primary DSF metabolite DTC, CuET otherwise was more toxic towards cancer cells, as evidenced by a dramatic drop of cell viability in 4T1-LG12 cells starting from 0.5 μM (Supplementary Figure S8B, P<0.05). Afterwards, the cytotoxicity of CuET/DIR NPs was determined. Figure 4A shows a significant dose-dependent tumor-killing efficacy in 4T1-LG12 cells upon CuET/DIR NPs from 0.2 to 0.8 μg/mL (P<0.05), and NIR laser irradiation further enhanced the tumor-killing efficacy of CuET/DIR NPs with 21-33% reduction of cell viability relative to those without NIR laser irradiation (Figure 4A, P<0.05). The enhanced tumor-killing efficacy should be mainly accountable to the intracellular localization of CuET with the aid of the nano-vehicles and local hyperthermia effects around the perinuclear region, as described above. Furthermore, the *in vivo* anticancer activity of CuET/DIR NPs was examined in animal model with xenotransplanted breast tumors derived from 4T1-LG12 cells, as established in our laboratory 37,38. The tissue distribution profiles indicated that liver, spleen and lung were the major organs for CuET/DIR NPs accumulation with much less amount in heart, kidney and blood, as characterized by Cu mass through ICP-MS (Figure 4B, P<0.05), in parallel to most nanomedicines 50-52. Based on these results (Figure 4B, P<0.05), the circulation time of CuET/DIR NPs was calculated to be approximately 48 h, which offered relatively long circulation time for nanodrugs to accumulate at the tumor sites. Strikingly, CuET/DIR NPs showed incredible inclination to accumulate in tumors with a comparable amount to that in the lung, and the accumulation increased over time from 12 to 48 h in line with the decline in the liver (Figure 4B, P<0.05). The preferential feature of tumor accumulation for CuET/DIR NPs offers an advantage to liberate drugs from the nanomedicines. Meanwhile, DIR fluorescence dye encapsulated in CuET/DIR NPs enabled the imaging of the tumors, which could visualize the localization of CuET/DIR NPs. Consistent with the ICP-MS results, CuET/DIR NPs exhibited appreciable localization in orthotopic primary tumors over time (Figure 4C-E, P<0.05). Additional single organ imaging further substantiated the massive accumulation of CuET/DIR NPs in tumors (Figure 4D).

The above findings encouraged us to interrogate the anticancer effects of CuET/DIR NPs *in vivo*. In the following, we thereby looked into tumor growth using the orthotopic model by transplanting 4T1-LG12 cells in mammary fat pads of BALB/c mice. In analogy to previous reports 29, CuET administration alone at 216 μg/Kg body weight could slow down tumor growth over the time course (Figure 4F, P<0.001). Of note, our effect of CuET administration alone showed a less extent than what has been described previously 29, and a few reasons were proposed to interpret this difference, including the administration protocol and total administration dose and other tumor features (*e.g.* heterogeneity within the tumor microenvironment and drug resistance 53-55). Without NIR laser irradiation, individual intravenous administration of CuET/DIR NPs did not show significant repression on tumor growth (Figure 4F-G). Importantly, the administration of CuET/DIR nanomedicines plus 808 nm laser irradiation substantially restrained tumor growth with no palpable tumors left on day 12 (Figure 4G). On this regard, on the one hand, CuET/DIR nanomedicines enhanced blood circulation time and tumor accumulation (Figure 4B, P<0.05). On the other hand, NIR laser triggered more liberation of CuET to attack the nucleus. By contrast, limited ablation of primary tumors was obtained if only dependent on the photothermal effects of DIR, as observed in the DIR NPs + NIR group relative to the CuET/DIR NPs + NIR group (Figure 4F, P<0.001), suggesting the indispensable role of NIR laser irradiation in initiating CuET-induced chemotherapy. Images of dissected tumors also corroborated these observations (Figure 4G). Nonetheless, no noticeable gross toxicity was defined in the animals upon these treatments, including physical activities and diet. And no significant alterations of body weight were found in mice upon diverse treatments compared to untreated control (Supplementary Figure S9). Plus, histological examination of an array of organs validated the excellent biosafety and biocompatibility of these nanomedicines (Supplementary Figure S10). Collectively, these results highlighted a pronounced synergistic thermo-chemotherapeutic capacity of CuET/DIR NPs to suppress primary breast cancer growth, where CuET-induced chemotherapy was triggered and subsequently reinforced by NIR laser irradiation.

**Figure 5:**
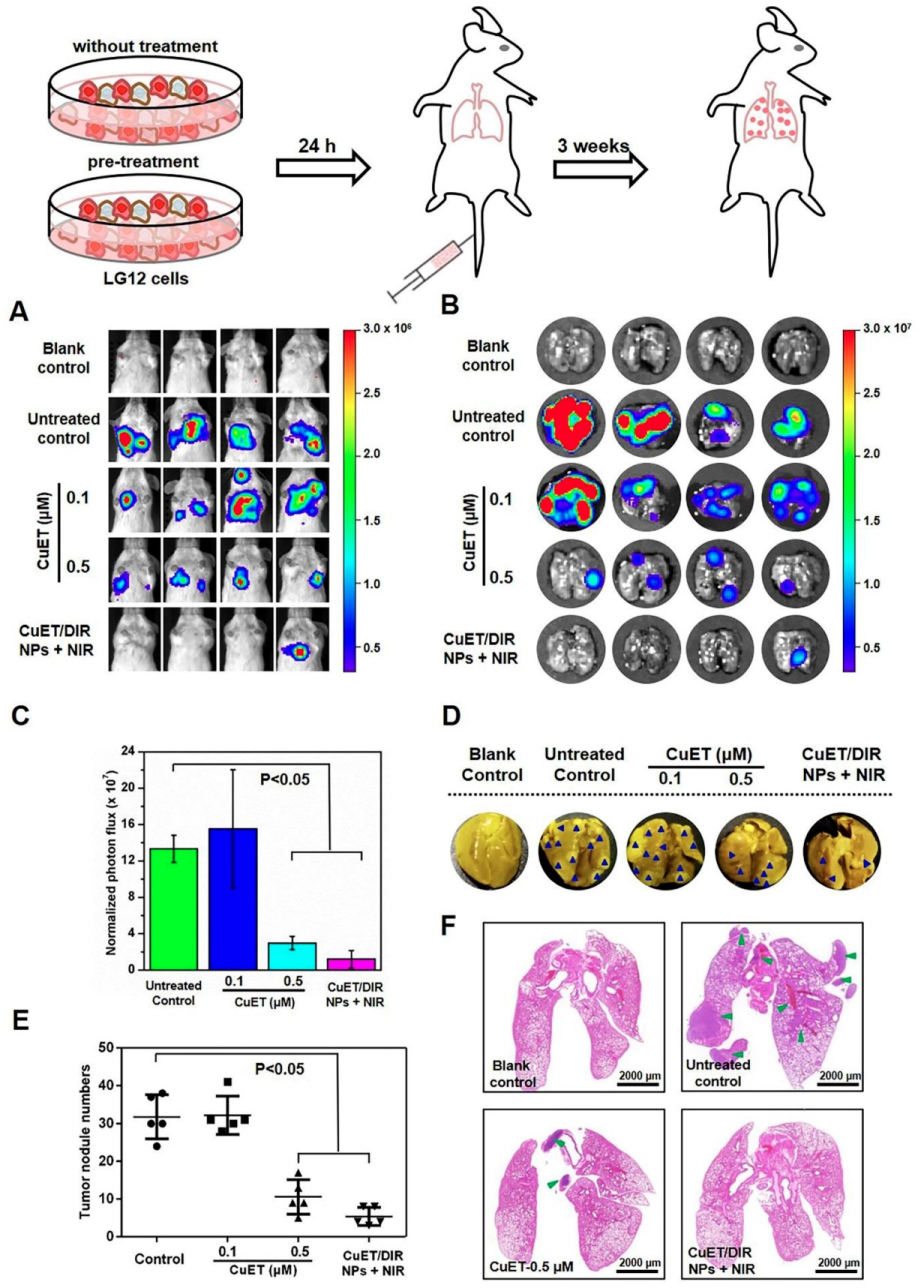
** CuET/DIR NPs undermined the intrinsic metastatic propensity of 4T1-LG12 cells to the lung.** (A) Bioluminescent signals at the lung sites in mice of 4T1-LG12 cell lung metastasis model upon different treatments. 4T1-LG12 cells were first pre-treated with CuET at 0.1 and 0.5 μM or CuET/DIR NPs containing 0.5 μM CuET together with NIR laser irradiation (808 nm, 2 W/cm^2^ for 5 min) for 24 h, and pre-treated cells were afterwards injected into mice through tail vein. Then, 3 weeks later, bioluminescence was imaged after intraperitoneal injection of D-luciferin at 75 mg/kg body weight (n=4). (B) Bioluminescent signal was separately imaged when mice were sacrificed. (C) Quantified bioluminescent signals on the lung sites in different groups. (D) Representative lung photographs from different groups. Dark blue arrowheads indicate metastatic tumors in the lungs. (E) Quantification of tumor nodules in the lungs from mice after injection of 4T1-LG12 cells with different pre-treatments including CuET and CuET/DIR NPs together with NIR laser irradiation (808 nm, 2 W/cm^2^ for 5 min) (n=4). (F) H&E staining images of lung sections from different group. Green arrowheads point at tumor nodules in lung sites.

Given that metastasis accounts for the majority of cancer deaths, substantial efforts are being invested in search of metastasis-specific therapeutic agents. Under this setting, metastatic behaviors of 4T1-LG12 cells were thus scrutinized upon treatment with CuET and CuET/DIR NPs. To better depict the anti-metastasis effects, a metastasis-specific mouse model was employed, where 4T1-LG12 cells were intravenously injected through tail vein and thereafter massive lung metastatic tumors were developed 37,38. Consistent with our previous reports 38, considerable lung metastasis was formed in untreated mice, as characterized by bioluminescent imaging of the animals and dissected lungs (Figure 5A-B). Although CuET pre-treatment at a lower dose (0.1 μM) did not elicit significant inhibition on metastatic tumors, higher-dose CuET (0.5 μM) otherwise incurred great repression on lung metastasis (Figure 5C, P<0.05). Intriguingly, CuET/DIR NP-mediated combinatory thermo-chemotherapy induced even greater repression on lung metastasis than CuET itself at the same dose (0.5 μM), as nearly no bioluminescent signaling could be visualized in CuET/DIR NP-treated mice (Figure 5A-B). To substantiate this finding, metastatic tumor nodules in the lungs were directly counted after fixing with Bouin's Fluid*.* As shown in Figure 5D-E, the number of lung nodules was reduced by 67% in the CuET group (at 0.5 μM), compared to untreated control, and more than 83% reduction was demonstrated in CuET/DIR NPs with NIR laser-treated mice relative to untreated mice (P<0.05), signifying the greater efficacy of CuET/DIR NP-conducted combined thermo-chemotherapy against metastasis. Additionally, histological examination with H&E staining confirmed the reduction of metastatic tumors in the lungs from mice upon treatment with CuET and CuET/DIR NPs together with NIR laser irradiation (Figure 5F).

**Figure 6:**
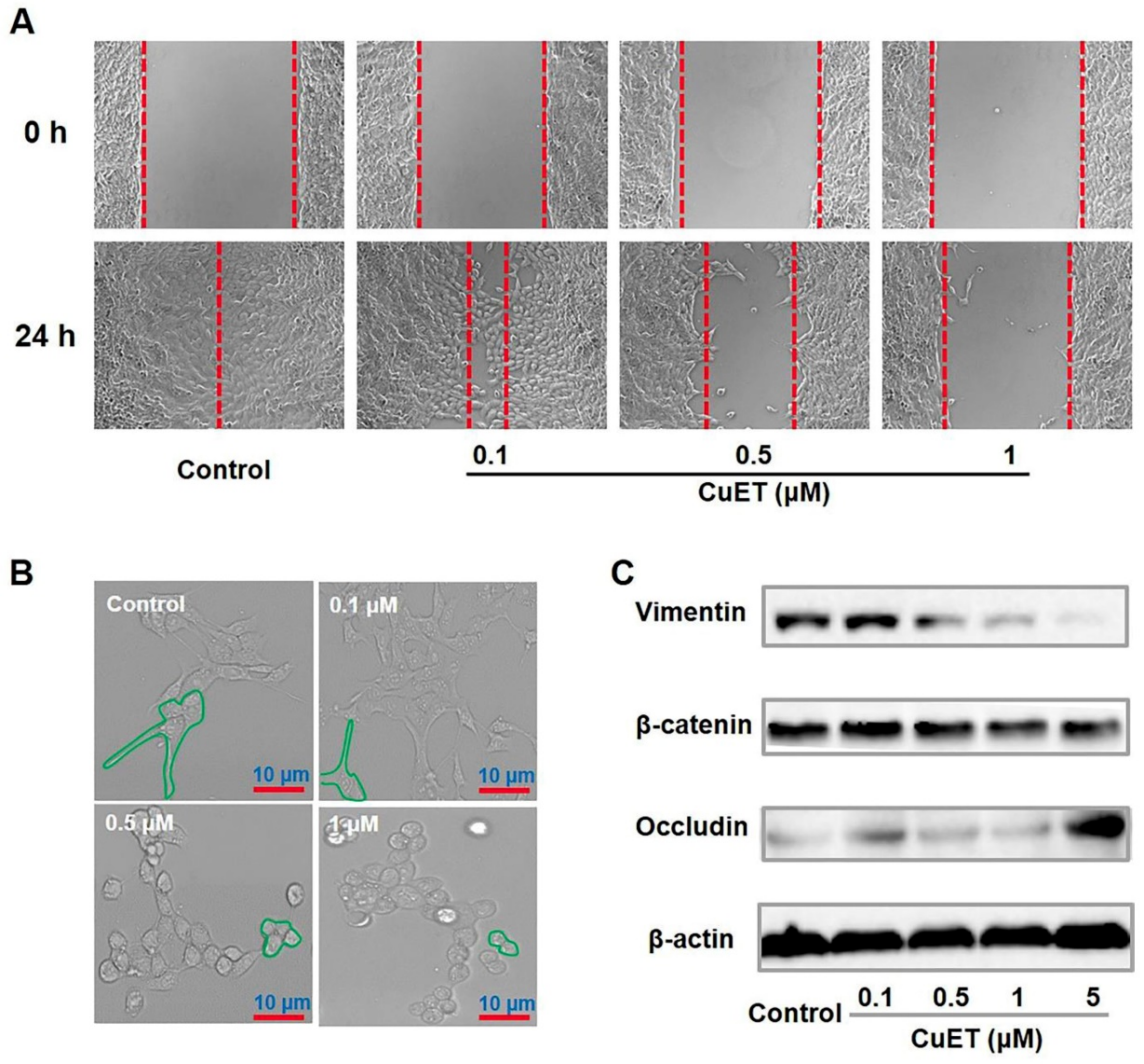
** Molecular mechanisms underlying CuET-induced repression on cancer metastatic behaviors.** (A) Representative images of wound-healing assay for 4T1-LG12 cells without or with CuET treatment at different concentrations for 24 h. (B) Morphological alterations of 4T1-LG12 cells upon CuET treatment at different concentrations for 24 h. Scale bars: 10 μm. (C) Protein levels of Vimentin, β-catenin and Occludin in 4T1-LG12 cells upon CuET at different concentrations for 6 h.

Afterwards, to inspect the molecular mechanisms underlying the anti-metastatic capability of CuET, cellular migration/invasion behaviors and the EMT program upon CuET were thoroughly probed. As shown in Figure 6A, CuET greatly repressed mobility/migration of 4T1-LG12 cells from 0.1 to 1 μM in a dose-dependent manner, as reflected by the wound-healing assay. To validate these results, the transwell assessment was carried out, and significant inhibition on cellular migration/invasion was found in cells treated with 0.1 μM CuET, as demonstrated by a dramatic drop of transmigrated cells relative to untreated cells (Supplementary Figure S11A). Cancer metastasis is a rather complex process involving a series of biological programs, of which EMT is generally believed to be the driving force for cancer cell transition from epithelial features into mesenchymal phenotypes 56. EMT would remarkably change the morphology of cancer cells with elevated propensity to metastasize to distant organs. As delineated in Figure 6B, the cellular morphology of 4T1-LG12 cells upon CuET at different concentrations was markedly altered from a “mesenchymal” shape to an “epithelial” shape, indicative of the reverse process of EMT, namely mesenchymal-epithelial transition (MET). Similar morphological changes were also observed in the parental 4T1 cells responding to CuET (Supplementary Figure S11B). To verify the inhibited EMT progress occurring in CuET-treated cells, a few representative surrogates were examined. Vimentin encodes an intermediate filament protein responsible for cell migration through integrating mechanical stimuli from outside and orchestrating the dynamics of cytoskeleton, and diminished vimentin level would fairly restrain tumor cell invasion and migration 57-59. Likewise, the Wnt/β-catenin signaling plays a crucial role in promoting metastasis through transcriptional activation of genes involved in EMT induction. Meanwhile, as an epithelial cell marker, occludin is an integral transmembrane protein at tight junctions, and increased occludin concentration would enhance cell-to-cell adhesion. As shown in Figure 6C, reduced levels of vimentin and β-catenin and reversely increased occludin, were found in 4T1-LG12 cells upon CuET in a dose-dependent manner. Together, these findings uncovered that CuET greatly compromised the EMT program to leash the metastatic behaviors of 4T1-LG12/4T1 cancer cells.

## Conclusions

In summary, the active form of DSF, an old alcohol-aversion drug, namely CuET, was synthesized and fabricated for cancer chemotherapeutics. Different from proposed mechanisms for CuET-conduced tumor-killing activity, the present work opens the wider caliber for the CuET-based anticancer drug design through developing an easy-to-do strategy in triggering light-responsive nuclear drug influx, resulting in DNA damage and cell apoptosis in breast cancer cells. Our CuET/DIR nanomedicines garnered a few advisable features in combatting tumors and metastases, including enhanced drug accumulation in the tumor, self-traceability for tumor bioimaging and photo-responsive release of laden anticancer drugs. Mechanistically, CuET/DIR nanomedicines function like “Trojan horse” to efficiently deliver the CuET drug inside the cell, and upon NIR laser irradiation, CuET would be liberated from the nanocomplexes to localize the nucleus, where the pro-apoptotic effects were induced (Supplementary Figure S6). And the NIR light-responsive nuclear delivery was designed to enhance the intranuclear intrusion. Furthermore, in contrast to previous anti-tumor growth only at the primary tumor, our CuET nanomedicines showed a considerable efficacy in diminishing breast cancer metastases to the lung through essentially undermining the intrinsic metastatic propensity of cancer cells dependent on inhibition on the EMT program. Additionally, our CuET/DIR NPs exhibited little toxicity to normal tissues, showing greater biocompatibility. These merits collectively endow our nanomedicines with a remarkably enhanced tumor-killing efficacy and great biocompatibility. Our findings undoubtedly will open up new avenues to investigate broader applications of DSF in cancer therapeutics, and will lead to critical insights into concomitant thermo-chemotherapy under a smart control mechanism to thwart primary tumor growth and invasive properties of metastatic cancer cells as well.

## Supplementary Material

Supplementary figures and tables.

## Abbreviations

DSF: disulfiram
DTC: diethyldithiocarbamate
CuET: (DTC)-Cu complex
PCM: phase-change material
NIR: near-infrared
EMT: epithelial-mesenchymal transition
ALDH: acetaldehyde dehydrogenase
NPL4: localization-4
DIR: DIR iodide
DSPE-PEG5000: 1,2-distearoyl-sn-glycero-3-phosphoethanolamine-N-[methoxy(polyethylene glycol)-5000
MTT: thiazolyl blue tetrazolium bromide
DMSO: dimethyl sulfoxide
FBS: fetal bovine serum
PBS: phosphate buffered saline
TEM: transmission electron microscope
SEM: scanning electron microscope
DLS: dynamic light scattering
ICP-MS: inductively coupled plasma mass spectrometry
EE: encapsulation efficiency
H&E: hematoxylin and eosin
CCK-8: cell counting kit-8
PI: propidium iodide
Abs: antibodies
BSA: albumin from bovine serum
DAPI: 4',6-diamidino-2-phenylindole
UPLC-MS/MS: ultra-performance liquid chromatography-tandem mass spectrometry
8-oxodG: 8-oxo-7,8-dihydro-2'-deoxyguanosine
PCMs: organic phase-change nanomaterials
MET: mesenchymal-epithelial transition

## References

[1] Bray F, Ferlay J, Soerjomataram I, Siegel RL, Torre LA, Jemal A (2018). Global cancer statistics 2018: GLOBOCAN estimates of incidence and mortality worldwide for 36 cancers in 185 countries. CA Cancer J Clin.

[2] Arruebo M, Vilaboa N, Saez-Gutierrez B, Lambea J, Tres A, Valladares M (2011). Assessment of the evolution of cancer treatment therapies. Cancers.

[3] Kreuter MW, Green MC, Cappella JN, Slater MD, Wise ME, Storey D (2007). Narrative communication in cancer prevention and control: a framework to guide research and application. Ann Behav Med.

[4] Eccles SA, Aboagye EO, Ali S, Anderson AS, Armes J, Berditchevski F (2013). Critical research gaps and translational priorities for the successful prevention and treatment of breast cancer. Breast Cancer Res.

[5] DeVita VT, Chu E (2008). A history of cancer chemotherapy. Cancer Res.

[6] ElBagoury M, Kotb M (2018). Chemotherapy over the years. J Pharm Sci Res.

[7] Crawford S (2013). Is it time for a new paradigm for systemic cancer treatment? Lessons from a century of cancer chemotherapy. Front Pharmacol.

[8] Seretny M, Currie GL, Sena ES, Ramnarine S, Grant R, MacLeod MR (2014). Incidence, prevalence, and predictors of chemotherapy-induced peripheral neuropathy: a systematic review and meta-analysis. Pain.

[9] Mansoori B, Mohammadi A, Davudian S, Shirjang S, Baradaran B (2017). The different mechanisms of cancer drug resistance: a brief review. Adv Pharm Bull.

[10] Oprea TI, Bauman JE, Bologa CG, Buranda T, Chigaev A, Edwards BS (2011). Drug repurposing from an academic perspective. Drug Discov Today Ther Strateg.

[11] Oprea TI, Mestres J (2012). Drug repurposing: far beyond new targets for old drugs. AAPS J.

[12] Strittmatter SM (2014). Overcoming drug development bottlenecks with repurposing: old drugs learn new tricks. Nat Med.

[13] Corsello SM, Bittker JA, Liu Z, Gould J, McCarren P, Hirschman JE (2017). The drug repurposing hub: a next-generation drug library and information resource. Nat Med.

[14] Pushpakom S, Iorio F, Eyers PA, Escott KJ, Hopper S, Wells A (2019). Drug repurposing: progress, challenges and recommendations. Nat Rev Drug Discov.

[15] Conticello C, Martinetti D, Adamo L, Buccheri S, Giuffrida R, Parrinello N (2012). Disulfiram, an old drug with new potential therapeutic uses for human hematological malignancies. Int J Cancer.

[16] Triscott J, Rose Pambid M, Dunn SE (2015). Concise review: bullseye: targeting cancer stem cells to improve the treatment of gliomas by repurposing disulfiram. Stem cells.

[17] Wuerth R, Thellung S, Bajetto A, Mazzanti M, Florio T, Barbieri F (2016). Drug-repositioning opportunities for cancer therapy: novel molecular targets for known compounds. Drug Discov Today.

[18] Ekinci E, Rohondia S, Khan R, Dou Q (2019). Repurposing disulfiram as an anti-cancer agent: updated review on literature and patents. Recent Pat Anticancer Drug Discov.

[19] Chuang MC, Yang YH, Tsai YH, Hsieh MJ, Lin YC, Lin CK (2018). Survival benefit associated with metformin use in inoperable non-small cell lung cancer patients with diabetes: a population-based retrospective cohort study. PloS One.

[20] Jian-Yu E, Graber JM, Lu SE, Lin Y, Lu-Yao G, Tan XL (2018). Effect of metformin and statin use on survival in pancreatic cancer patients: a systematic literature review and meta-analysis. Curr Med Chem.

[21] Kim JS, Turbov J, Rosales R, Thaete LG, Rodriguez GC (2019). Combination simvastatin and metformin synergistically inhibits endometrial cancer cell growth. Gynecol Oncol.

[22] Iljin K, Ketola K, Vainio P, Halonen P, Kohonen P, Fey V (2009). High-throughput cell-based screening of 4910 known drugs and drug-like small molecules identifies disulfiram as an inhibitor of prostate cancer cell growth. Clin Cancer Res.

[23] Cvek B, Milacic V, Taraba J, Dou QP (2008). Ni (II), Cu (II), and Zn (II) diethyldithiocarbamate complexes show various activities against the proteasome in breast cancer cells. J Med Chem.

[24] Chen D, Cui QC, Yang H, Dou QP (2006). Disulfiram, a clinically used anti-alcoholism drug and copper-binding agent, induces apoptotic cell death in breast cancer cultures and xenografts via inhibition of the proteasome activity. Cancer Res.

[25] Yip N, Fombon I, Liu P, Brown S, Kannappan V, Armesilla A (2011). Disulfiram modulated ROS-MAPK and NFκB pathways and targeted breast cancer cells with cancer stem cell-like properties. Br J Cancer.

[26] Liu P, Brown S, Channathodiyil P, Kannappan V, Armesilla AL, Darling JL (2013). Reply: cytotoxic effect of disulfiram/copper on human glioblastoma cell lines and ALDH-positive cancer-stem-like cells. Br J Cancer.

[27] Zhang X, Hu P, Ding SY, Sun T, Liu L, Han S (2019). Induction of autophagy-dependent apoptosis in cancer cells through activation of ER stress: an uncovered anti-cancer mechanism by anti-alcoholism drug disulfiram. Am J Cancer Res.

[28] Bu W, Wang Z, Meng L, Li X, Liu X, Chen Y (2019). Disulfiram inhibits epithelial-mesenchymal transition through TGFβ-ERK-Snail pathway independently of Smad4 to decrease oral squamous cell carcinoma metastasis. Cancer Manag Res.

[29] Skrott Z, Mistrik M, Andersen KK, Friis S, Majera D, Gursky J (2017). Alcohol-abuse drug disulfiram targets cancer via p97 segregase adaptor NPL4. Nature.

[30] Santini C, Pellei M, Gandin V, Porchia M, Tisato F, Marzano C (2014). Advances in copper complexes as anticancer agents. Chem Rev.

[31] Denoyer D, Masaldan S, La Fontaine S, Cater MA (2015). Targeting copper in cancer therapy:'Copper That Cancer'. Metallomics.

[32] Bhattacharjee A, Chakraborty K, Shukla A (2017). Cellular copper homeostasis: current concepts on its interplay with glutathione homeostasis and its implication in physiology and human diseases. Metallomics.

[33] Wei Y, Zhu N, Lavoie M, Wang J, Qian H, Fu Z (2014). Copper toxicity to phaeodactylum tricornutum: a survey of the sensitivity of various toxicity endpoints at the physiological, biochemical, molecular and structural levels. Biometals.

[34] Xiao J, Chen S, Yi J, Zhang HF, Ameer GA (2017). A cooperative copper metal-organic framework-hydrogel system improves wound healing in diabetes. Adv Funct Mater.

[35] Guo L, Panderi I, Yan DD, Szulak K, Li Y, Chen YT (2013). A comparative study of hollow copper sulfide nanoparticles and hollow gold nanospheres on degradability and toxicity. ACS Nano.

[36] Zhu C, Huo D, Chen Q, Xue J, Shen S, Xia Y (2017). A eutectic mixture of fatural fatty acids can serve as the gating material for near-infrared-triggered drug release. Adv Mater.

[37] Guo W, Zhang S, Liu S (2015). Establishment of a novel orthotopic model of breast cancer metastasis to the lung. Oncol Rep.

[38] Wang S, Shao J, Li Z, Ren Q, Yu XF, Liu S (2019). Black phosphorus-based multimodal nanoagent: showing targeted combinatory therapeutics against cancer metastasis. Nano Lett.

[39] Liu S, Goldstein RH, Scepansky EM, Rosenblatt M (2009). Inhibition of rho-associated kinase signaling prevents breast cancer metastasis to human bone. Cancer Res.

[40] Zhang J, Wang S, Gao M, Li R, Liu S (2018). Multihierarchically profiling the biological effects of various metal-based nanoparticles in macrophages under low exposure doses. ACS Sustain Chem Eng.

[41] Jen-La Plante I, Zeid TW, Yang P, Mokari T (2010). Synthesis of metal sulfide nanomaterials via thermal decomposition of single-source precursors. J Mater Chem.

[42] Wehbe M, Anantha M, Shi M, Leung AW, Dragowska WH, Sanche L (2017). Development and optimization of an injectable formulation of copper diethyldithiocarbamate, an active anticancer agent. Int J Nanomedicine.

[43] Marengo A, Forciniti S, Dando I, Dalla Pozza E, Stella B, Tsapis N (2019). Pancreatic cancer stem cell proliferation is strongly inhibited by diethyldithiocarbamate-copper complex loaded into hyaluronic acid decorated liposomes. Biochim Biophys Acta Gen Subj.

[44] Zhao P, Wang Y, Kang X, Wu A, Yin W, Tang Y (2018). Dual-targeting biomimetic delivery for anti-glioma activity via remodeling the tumor microenvironment and directing macrophage-mediated immunotherapy. Chem Sci.

[45] Tesson M, Anselmi G, Bell C, Mairs R (2017). Cell cycle specific radiosensitisation by the disulfiram and copper complex. Oncotarget.

[46] Koh HK, Seo SY, Kim JH, Kim HJ, Chie EK, Kim SK (2019). Disulfiram, a re-positioned aldehyde dehydrogenase inhibitor, enhances radiosensitivity of human glioblastoma cells in vitro. Cancer Res Treat.

[47] Chen X, Zhang X, Guo Y, Zhu YX, Liu X, Chen Z (2019). Smart supramolecular “Trojan Horse”-inspired nanogels for realizing light-triggered nuclear drug influx in drug-resistant cancer cells. Adv Funct Mater.

[48] Gao G, Jiang YW, Jia HR, Sun W, Guo Y, Yu XW (2019). From perinuclear to intranuclear localization: a cell-penetrating peptide modification strategy to modulate cancer cell migration under mild laser irradiation and improve photothermal therapeutic performance. Biomaterials.

[49] Guo Y, Zhang X, Sun W, Jia HR, Zhu YX, Zhang X (2019). Metal-phenolic network-based nanocomplexes that evoke ferroptosis by apoptosis: promoted nuclear drug influx and reversed drug resistance of cancer. Chem Mater.

[50] Wu W, Yu L, Jiang Q, Huo M, Lin H, Wang L (2019). Enhanced tumor-specific disulfiram chemotherapy by in situ Cu^2+^ chelation-initiated nontoxicity-to-toxicity transition. J Am Chem Soc.

[51] Tang S, Chen M, Zheng N (2015). Multifunctional ultrasmall Pd nanosheets for enhanced near-infrared photothermal therapy and chemotherapy of cancer. Nano Res.

[52] Zhou M, Zhang R, Huang M, Lu W, Song S (2010). Melancon MP, et al. A chelator-free multifunctional [64Cu] CuS nanoparticle platform for simultaneous micro-PET/CT imaging and photothermal ablation therapy. J Am Chem Soc.

[53] Trédan O, Galmarini CM, Patel K, Tannock IF (2007). Drug resistance and the solid tumor microenvironment. J Natl Cancer Inst.

[54] Manzoor AA, Lindner LH, Landon CD, Park JY, Simnick AJ, Dreher MR (2012). et al. Overcoming limitations in nanoparticle drug delivery: triggered, intravascular release to improve drug penetration into tumors. Cancer Res.

[55] Andresen TL, Jensen SS, Jørgensen K (2005). Advanced strategies in liposomal cancer therapy: problems and prospects of active and tumor specific drug release. Prog Lipid Res.

[56] Luan M, Chang J, Pan W, Chen Y, Li N, Tang B (2018). Simultaneous fluorescence visualization of epithelial-mesenchymal transition and apoptosis processes in tumor cells for evaluating the impact of epithelial-mesenchymal transition on drug efficacy. Anal Chem.

[57] Vuoriluoto K, Haugen H, Kiviluoto S, Mpindi JP, Nevo J, Gjerdrum C (2011). Vimentin regulates EMT induction by slug and oncogenic H-Ras and migration by governing Axl expression in breast cancer. Oncogene.

[58] Chaw SY, Majeed AA, Dalley AJ, Chan A, Stein S, Farah CS (2012). Epithelial to mesenchymal transition (EMT) biomarkers-E-cadherin, beta-catenin, APC and Vimentin-in oral squamous cell carcinogenesis and transformation. Oral Oncol.

[59] Ivaska J (2011). Vimentin: central hub in EMT induction?. Small GTPases.

