# Supplementary material for: Diethyldithiocarbamate-copper nanocomplex reinforces disulfiram chemotherapeutic efficacy through light-triggered nuclear targeting
Source: Theranostics. 2020 May 16;10(14):6384–98. doi: 10.7150/thno.45558 (PMC7255023; doi:10.7150/thno.45558)
Supplement: Supplementary file 1 — Supplementary figures and tables. [file thnov10p6384s1.pdf]

Supplementary Information

Diethyldithiocarbamate-copper nanocomplex reinforces disulfiram chemotherapeutic efficacy through light-triggered nuclear targeting

Liting Ren[1,2,#], Wenya Feng[1,3,#], Jie Shao[1,3], Juan Ma[1,3], Ming Xu[1,3], Ben-Zhan Zhu[1,3], Nanfeng Zheng[2], Sijin Liu[1,3,*]

1. State Key Laboratory of Environmental Chemistry and Ecotoxicology, Research Center for Eco-Environmental Sciences, Chinese Academy of Sciences, Beijing, 100085, China.
2. State Key Laboratory for Physical Chemistry of Solid Surfaces, Collaborative Innovation Center of Chemistry for Energy Materials, and National & Local Joint Engineering Research Center for Preparation Technology of Nanomaterials, College of Chemistry and Chemical Engineering, Xiamen University, Xiamen, 361005, China.
3. University of Chinese Academy of Sciences, Beijing, 100049, China.

#: these authors equally contribute to this work.

*: correspondence to Sijin Liu, Ph.D, email: sjliu@rcees.ac.cn

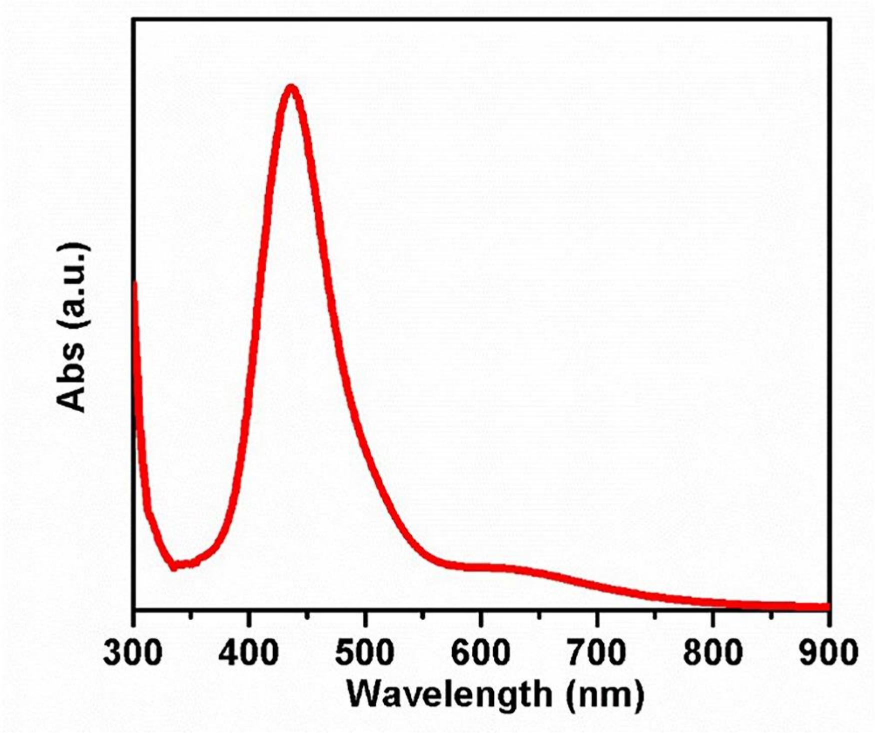

Figure S1. UV-vis absorption spectra of CuET in dichloromethane solution.

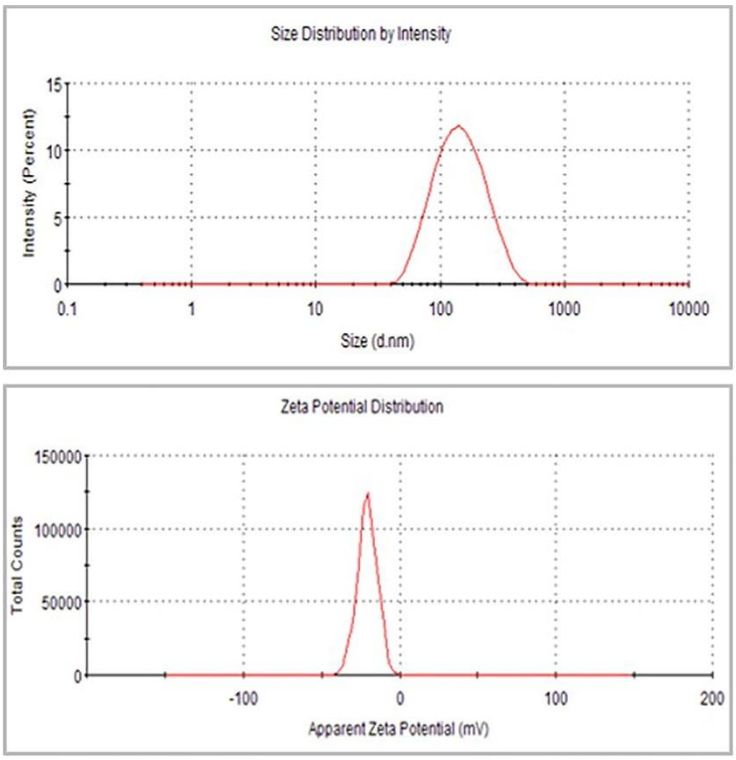

Figure S2. Particle size distribution measured by DLS (the upper panel) and Zeta potential (the lower panel) of CuET/DIR NPs.

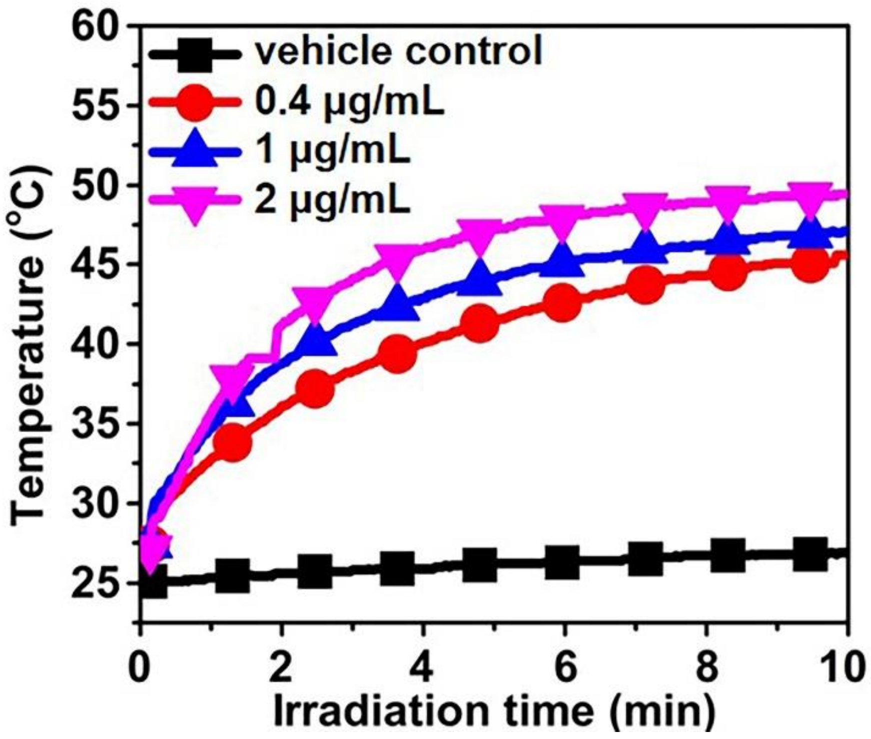

Figure S3. Temperature elevation of CuET/DIR NPs solutions at different concentrations under 808 nm laser irradiation at 2 W/cm^2 power density for 10 min.

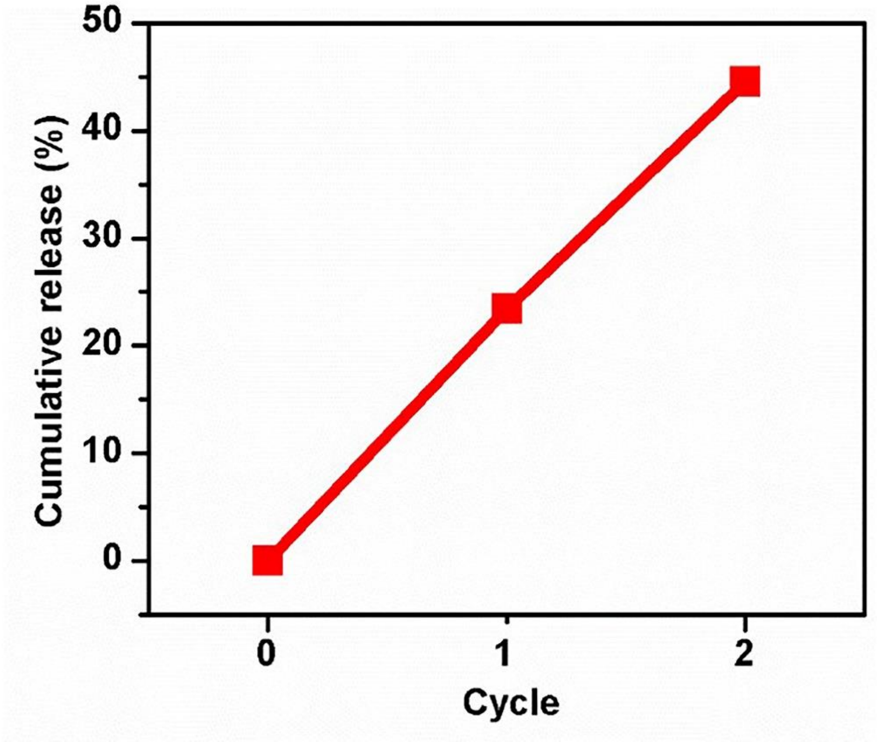

Figure S4. Cumulative CuET release from CuET/DIR NPs (containing 0.4 μg/mL CuET) upon repeated cycles of laser irradiation (808 nm, 2 W/cm^2).

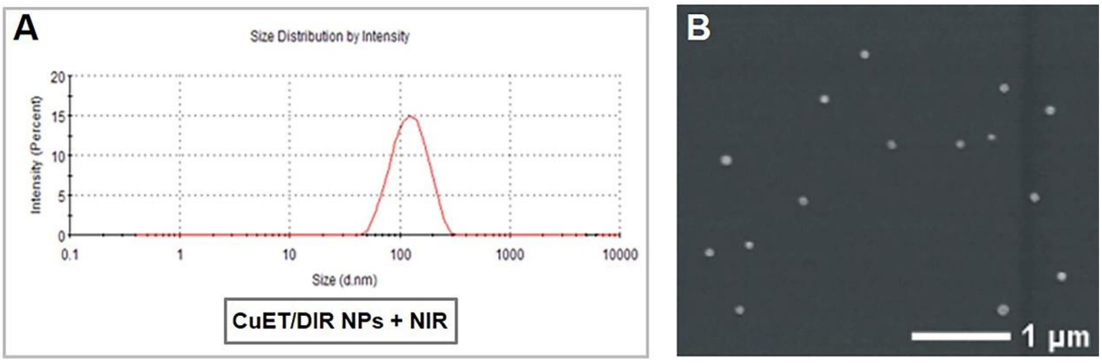

Figure S5. Characterization of CuET/DIR NPs after NIR laser irradiation. (A) Particle size distribution measured by DLS and (B) representative SEM image of CuET/DIR NPs after irradiation with NIR laser (808 nm, 2 W/cm^2) for 5 min.

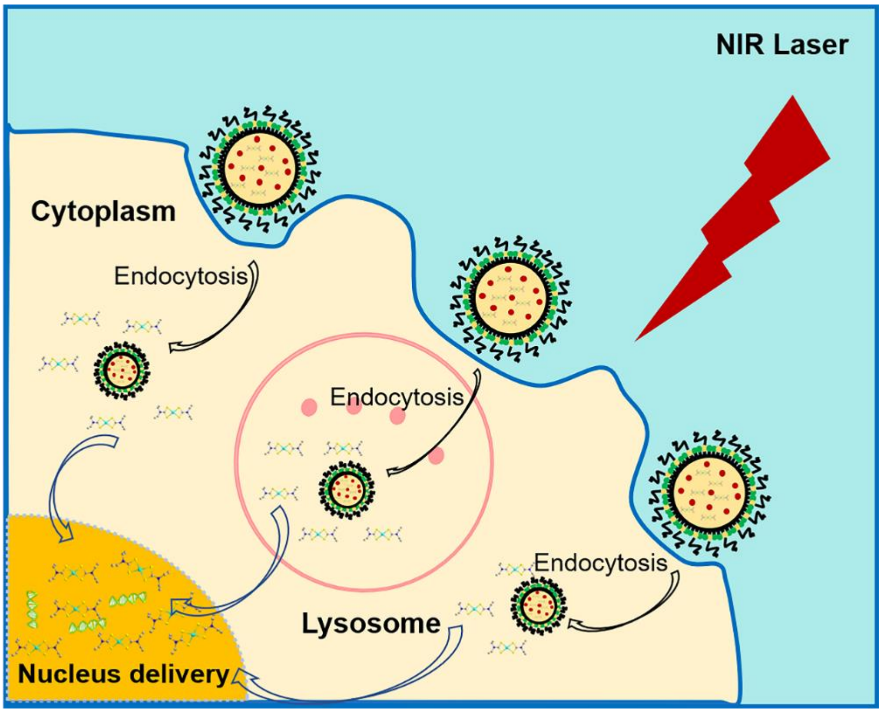

Figure S6. A schematic illustrates CuET/DIR nanomedicines that behave like “Trojan horse” to enhance the cellular uptake and nuclear delivery of CuET.

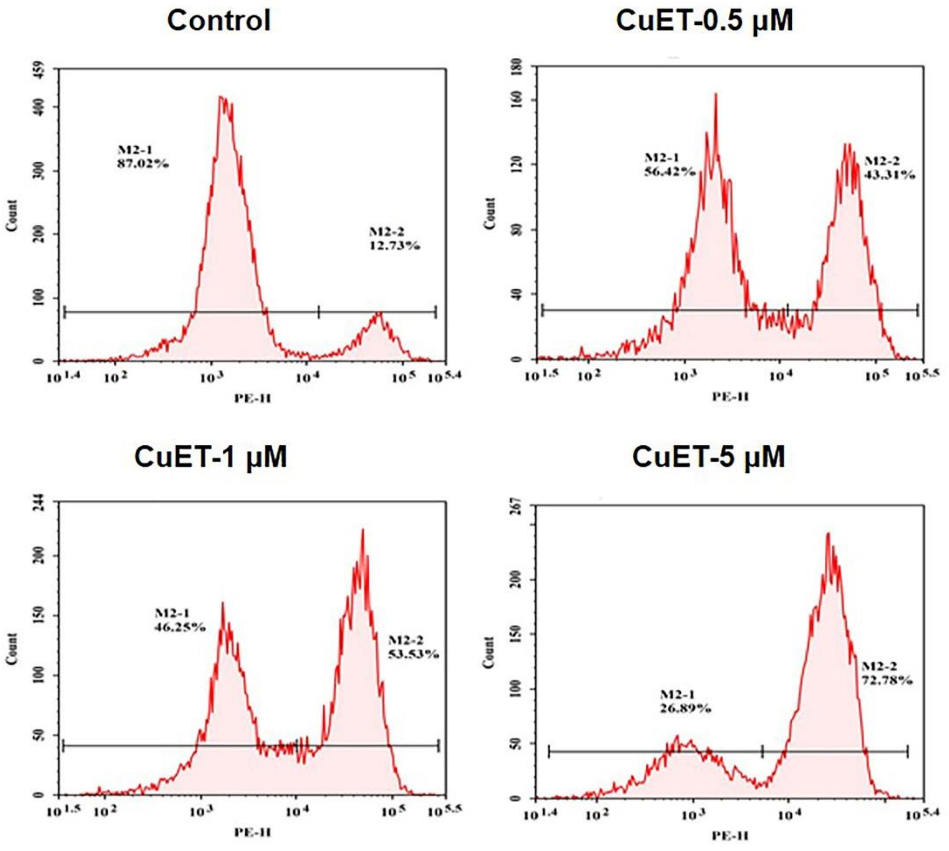

Figure S7. Flow cytometry analysis using propidium iodide (PI) single staining in 4T1-LG12 cells upon CuET at different concentrations for 24 h.

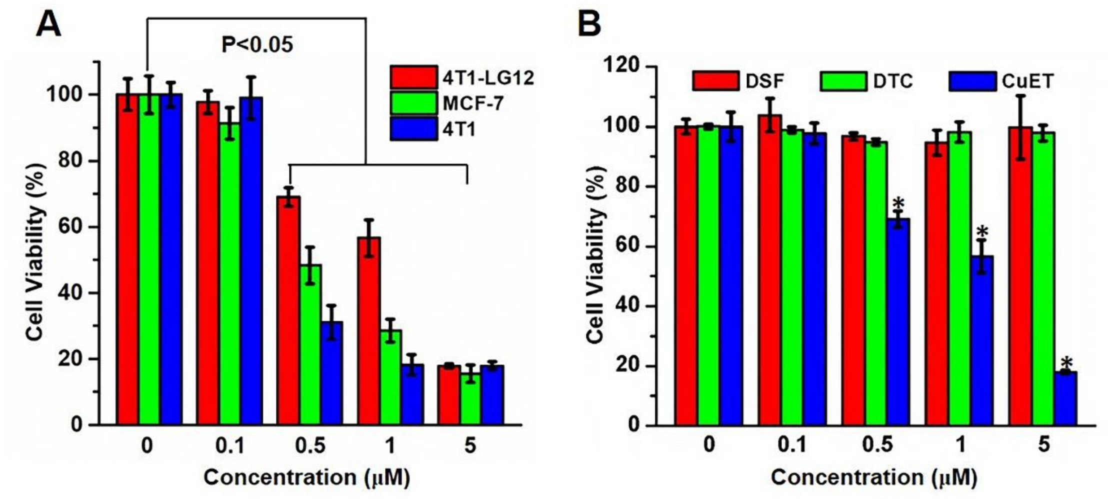

Figure S8. Cell viability assessment. (A) Cell viability of various cancer cells including human breast cancer cell line MCF-7, 4T1 (parental line) and its subline 4T1-LG12, upon CuET at different concentrations for 24 h (n=4). (B) Cell viability of 4T1-LG12 upon DSF, DTC and CuET at different concentrations for 24 h (n=4).

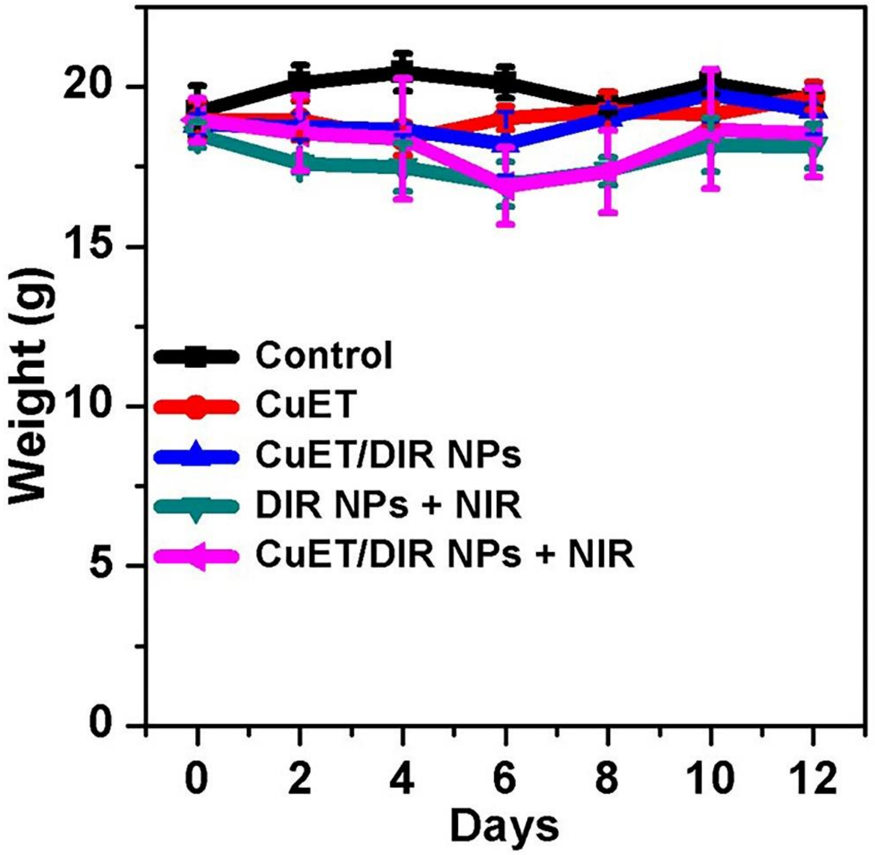

Figure S9. Changes in body weight of the mice during treatment.

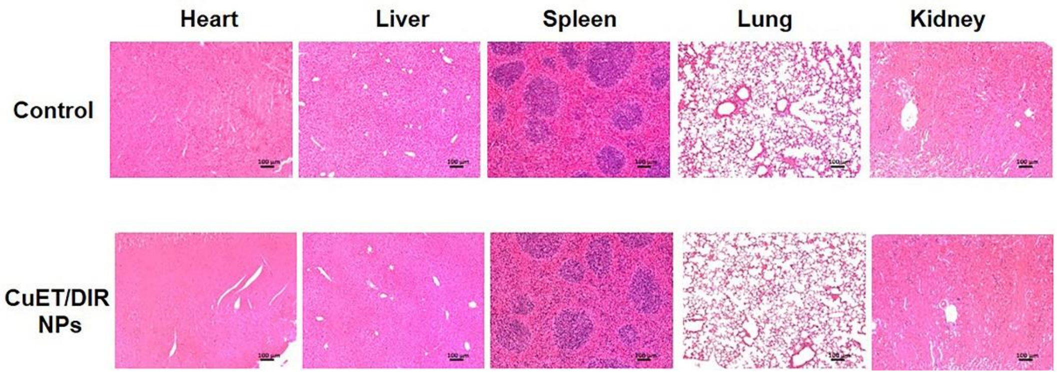

Figure S10. Histological examination with H&E staining of heart, liver, spleen, lung and kidney sections from mice post various treatments for 48 h.

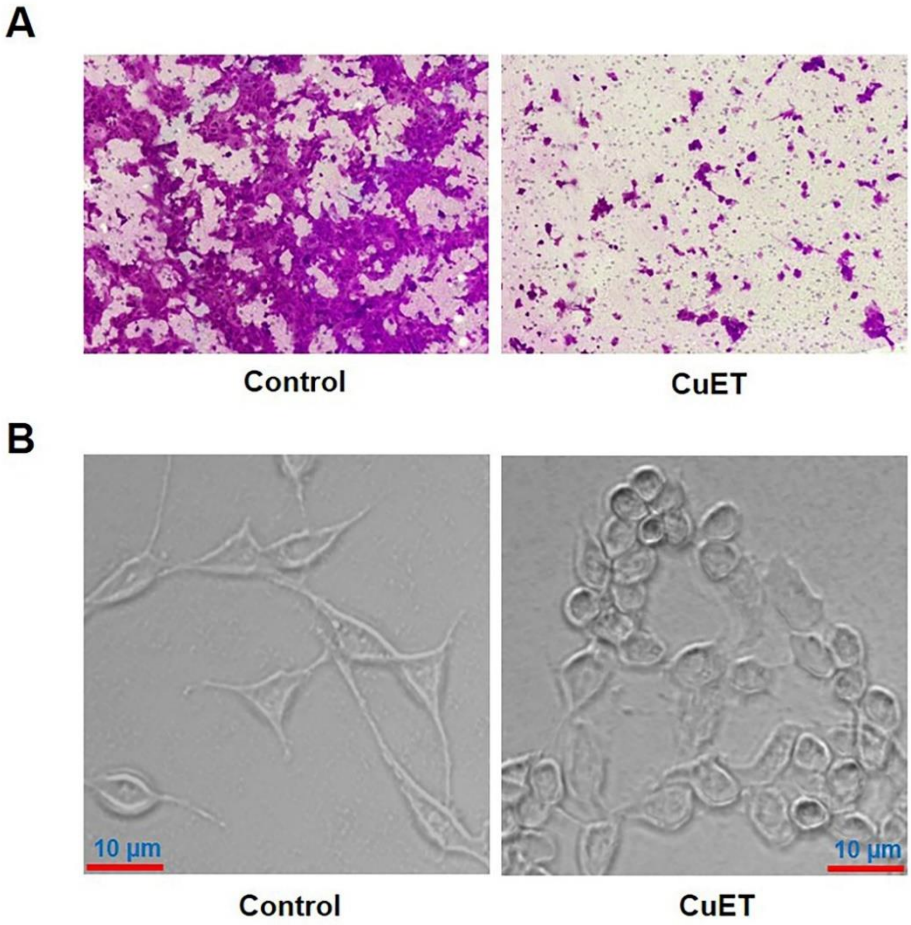

Figure S11. (A) Transwell migration/invasion assay for 4T1-LG12 cells without or with CuET treatment at 0.1μM for 24 h. (B) The effects of CuET at 0.5 μM on cell morphology of parental 4T1 cells after incubation for 24 h. Scale bars: 10 μm.
